# *RRAGD* p.(Ser76Leu) Variant Causes Dysregulated Expression of Muscle Development and Cytoskeleton Genes in Cardiomyocytes

**DOI:** 10.1096/fj.202501099RR

**Published:** 2026-06-22

**Authors:** Anastasia Adella, Sara B. van Katwijk, Pieter A. Leermakers, Willem B. van Ham, Hesther de Ruiter, Judita Ilgutytė, Suzanne Hendrickx, Levi Nijland, Teun P. de Boer, Eva van Rooij, Joost G. J. Hoenderop, Jeroen H. F. de Baaij

**Affiliations:** ^1^ Department of Medical BioSciences Radboudumc Nijmegen the Netherlands; ^2^ Department of Medical Physiology University Medical Center Utrecht Utrecht the Netherlands; ^3^ Hubrecht Institute, Royal Netherlands Academy of Arts and Sciences (KNAW) and University Medical Center Utrecht Utrecht the Netherlands; ^4^ Division Heart and Lungs, Department of Cardiology University Medical Center Utrecht Utrecht the Netherlands

**Keywords:** ADKH‐*RRAGD*, cardiomyocytes, dilated cardiomyopathy, mTORC1, *RRAGD*

## Abstract

Autosomal dominant kidney hypomagnesemia with *RRAGD* variants (ADKH‐*RRAGD*) is a hereditary disorder characterized by kidney tubulopathy and dilated cardiomyopathy (DCM). RagD, encoded by the *RRAGD* gene, is a small GTPase involved in activating the mechanistic target of rapamycin complex 1 (mTORC1) by amino acids. Although several gain‐of‐function variants in the *RRAGD* gene have been identified, their contributions to DCM remain unclear. Here, we hypothesize that these *RRAGD* variants induce mTORC1 overactivation, thereby contributing to the manifestation of DCM. To investigate this, we established T‐REx HeLa cell lines that overexpress the *RRAGD* p.(Ser76Leu) or the wild‐type (WT) variant to assess the effects on mTORC1 signaling. Additionally, we developed the first cellular model of ADKH‐*RRAGD* utilizing genetically edited human‐induced pluripotent stem cell‐derived cardiomyocytes (hiPSC‐CMs) that express the mutated variant. Our data indicate that the *RRAGD* p.(Ser76Leu) variant maintains the phosphorylation of mTORC1 targets (i.e., S6K, 4E‐BP1, and TFEB) during amino acid starvation, in contrast to *RRAGD* WT in T‐REx HeLa cells. The pharmacological inhibition of mTOR with Torin1 reversed these changes. In 2D‐cultured *RRAGD* ^WT/p.(Ser76Leu)^ hiPSC‐CMs, mTORC1 remained responsive to amino acid starvation. Results from bulk RNA sequencing showed an upregulation of pathways associated with cytoskeletal organization and a downregulation of muscle development in *RRAGD* ^WT/p.(Ser76Leu)^ hiPSC‐CMs. Moreover, a prolonged duration of Ca^2+^ transients was observed in the mutant cardiomyocytes. Altogether, our data demonstrate that gain‐of‐function variants in *RRAGD* cause mTORC1 activation in T‐REx HeLa cells. Consequently, cardiomyocytes develop impaired intracellular Ca^2+^ clearance and activation of transcriptional programs, suggesting dedifferentiation.

## Graphical abstract

Under normal conditions, RagD and mTORC1 respond to amino acid changes. Upon stimulation, active mTORC1 phosphorylates S6K, 4E‐BP1, and TFEB. In response to starvation, mTORC1 is inactive. Our data indicate that the *RRAGD* p.(Ser76Leu) variant maintains the phosphorylation of mTORC1 targets during starvation in T‐REx HeLa cells. While mTORC1 remained responsive to starvation in *RRAGD* ^WT/p.(Ser76Leu)^ hiPSC‐CMs. Moreover, prolonged duration of Ca^2+^ transients and dysregulation of pathways associated with muscle development and cytoskeletal organization were observed in *RRAGD* ^WT/p.(Ser76Leu)^ hiPSC‐CMs.

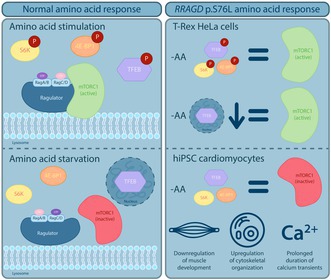

## 1 Introduction

Gain‐of‐function variants in Ras‐related GTPase D (RRAGD) are causative for kidney tubulopathy and dilated cardiomyopathy (DCM) [1, 2]. In patients with pathogenic *RRAGD* variants, cardiomyopathies have been reported in approximately 50% of all cases, requiring heart transplantation in 41% of these cases [3]. Other cardiac defects include ventricular arrhythmias, myocardial infarction, and excessive apical trabeculations in a subset of patients [2, 4]. Within the spectrum of *RRAGD* variants, the most prevalent variant, p.(Ser76Leu), is present in a mutational hotspot that is associated with cardiac defects [1]. As 82% of patients carrying this variant developed cardiomyopathy [1, 4].

*RRAGD*, encoding the RagD protein, belongs to the family of rag GTPases that act as intracellular amino acid sensors. RagD is involved in activating the mechanistic target of rapamycin complex 1 (mTORC1) by amino acids. The Rag GTPases recruit mTORC1 to the lysosomal surface, where it phosphorylates its downstream targets [5, 6]. RagC and RagD form heterodimeric complexes with RagA or RagB. Upon amino acid‐induced activation, RagA and RagB bind GTP, whereas RagC and RagD are GDP‐bound [7, 8, 9, 10, 11]. After mTORC1 is activated at the lysosomal surface, mTORC1 phosphorylates canonical substrates such as the S6 kinase (S6K) and the eukaryotic initiation factor 4e binding protein 1 (4E‐BP1), and the non‐canonical mTORC1 substrates such as the member of the microphthalmia/transcription factor E (MiT/TFE) family, the transcription factor EB (TFEB) [12, 13, 14, 15, 16, 17, 18]. To date, pathogenic *RRAGD* variants have been demonstrated to disturb both canonical and non‐canonical mTORC1 pathways. In HEK293 cells, *RRAGD* variants resulted in constitutive activation of S6K in the absence of amino acids [1]. Moreover, Sambri et al. reported overactivation of only the non‐canonical mTORC1 pathway using TFEB as their main read‐out [2].

The regulation of mTOR is important in maintaining cardiac function and structure, both under normal conditions and during adaptation to stress (reviewed in [19, 20]). On the contrary, it has also been shown that partial inhibition of mTORC1 can reduce cardiovascular damage in cardiomyopathies. Suppression of mTOR activity has improved cardiac structure and functions in cell and animal models of DCM [19, 20]. For example, deletion of *Mtor* in mouse cardiomyocytes suppressed proliferation at early embryonic stages [21]. In adult mice, inducible *Mtor* deletion from the heart caused DCM [22]. The role of Rag GTPases in heart has also been demonstrated. *Rraga* and *Rragb* KO mice develop cardiac hypertrophy [23]. Additionally, gain‐of‐function variants in *rragc* cause a DCM‐like phenotype in cells and zebrafish [24, 25]. More recently, we showed that the *RRAGD* p.(Ser76Leu) and p.(Pro119Arg) variants led to cardiac dysfunctions in zebrafish embryos [26]. Although the full molecular mechanisms remain to be elucidated, multiple studies point towards reduced TFEB activity [2, 23, 24, 25]. Indeed, restoration of TFEB function rescued the *rragc‐*induced cardiac dysfunction in zebrafish [24].

In this study, we examined the cellular consequences of the *RRAGD* p.(Ser76Leu) variant in cardiomyocytes. We hypothesized that the *RRAGD* p.(Ser76Leu) variant leads to constitutive activation of mTORC1, which disrupts cardiomyocyte cellular processes and contributes to the DCM observed in patients. To investigate how this variant affects mTORC1 signaling, we utilized stable T‐REx HeLa cell lines overexpressing either *RRAGD* wild‐type (WT) or the p.(Ser76Leu) variant. Subsequently, we validated our findings in a more physiologically relevant model using a human‐induced pluripotent stem cell‐derived cardiomyocyte (hiPSC‐CM) model carrying a heterozygous p.(Ser76Leu) variant. Finally, using the hiPSC‐CMs, we performed electrophysiological assays and bulk RNA sequencing to systematically analyze the functional consequences of the variant.

## 2 Materials and Methods

### 2.1 Cloning and Plasmids

Human *RRAGD* construct (VectorBuilder GmbH, Neu‐Isenburg, Germany (vector ID: VB171030‐1104zuv; vectorbuilder.com)) was subcloned into the pCINeo IRES mCherry expression vector using AgeI and EcoRI restriction sites. *RRAGD* c.227C>T (p.(Ser76Leu)) mutation was introduced using the QuikChange II XL site‐directed mutagenesis kit (Agilent Technologies, California, USA). To make the T‐REx HeLa stable cell lines, *RRAGD* WT and p.(Ser76Leu) constructs were subcloned into the pcDNA5/FRT/TO eGFP expression vector using the KpnI and XhoI restriction sites. pcDNA3.1‐TFEB‐WT‐MYC was a gift from James Brugarolas (Addgene plasmid #99955; RRID:Addgene_99955) [27]. For CRISPR‐Cas9, gRNAs were subcloned into the pSpCas9(BB)‐2A‐GFP (PX458) vector (Addgene plasmid #48138; RRID:Addgene_48138). All primers and oligonucleotides used are available in Table S1.

### 2.2 Antibodies

Primary antibodies from Cell Signaling Technology (Massachusetts, USA): Akt (#4691, WB: 1:1000), p‐Akt^Thr308^ (#9275, WB: 1:500), p70‐S6K (#2708, WB: 1:1000), p‐p70‐S6K^Thr389^ (#9234, WB: 1:1000), TFEB (#4240, WB: 1:1000, ICC: 1:200), p‐TFEB^Ser211^ (#37681, WB: 1:1000), 4E‐BP1 (#9452, WB: 1:1000), p‐4E‐BP1^Thr37/46^ (#2855, WB: 1:1000). From Novius Biologicals (Colorado, USA): RagD (#NBP2‐32106, WB: 1:2000, ICC: 1:200). From Developmental Studies Hybridoma Bank (DSHB, Iowa City, USA): LAMP1 (AB_2296838 clone H4A3, ICC: 1:300). From Sigma‐Aldrich (Missouri, USA): ACTN2 (#A7732, ICC: 1:400, FACS: 1:1000), GFP (#G154, WB: 1:5000), and TAZ (#HPA007415, WB: 1:1000). From Invitrogen (Vilnius, Lithuania): GAPDH (#AM4300, WB: 1:4000). From Santa Cruz Biotechnology (Texas, USA): Vinculin (#sc‐25336, WB: 1:1000) and p‐TAZ^Ser89^ (#sc‐17610, WB: 1:1000). From Bethyl Laboratories (Texas, USA): TFEB (A303‐673A, ICC: 1:200). From Abcam (Cambridge, UK): cTnT (#45932, FACS: 1:1000). For immunoblotting, peroxidase‐ (PO) conjugated secondary antibodies were used. From Jackson ImmunoResearch Europe Ltd. (Exeter, UK): PO‐conjugated anti‐IgG mouse (#145‐515‐035, 1:10 000). From Merck Life Sciences (Amsterdam, the Netherlands): PO‐conjugated anti‐IgG rabbit (#A4914, 1:10 000) was used. For immunocytochemistry & FACS experiments, secondary antibodies from Thermo Fisher Scientific (Orlando, USA): anti‐IgG rabbit conjugated to Alexa Fluor 488 (#A11008, ICC: 1:300, FACS: 1:1000) and Alexa Fluor 594 (#A11012, ICC: 1:300), anti‐IgG mouse conjugated to Alexa Fluor 488 (#A11029, ICC: 1:300, FACS: 1:1000), and anti‐IgG mouse conjugated to Alexa Fluor 594 (#A11005, ICC: 1:300).

### 2.3 Cell Culture

T‐REx HeLa cells (RRID:CVCL_D587) and HEK293 cells (RRID:CVCL_0045) were cultured in culture medium (Dulbecco's Modified Eagle Medium (DMEM) containing 25 mM Hepes, 4.5 g/L glucose, and 4 mM L‐glutamine (#42430082, Thermo Fisher Scientific, California, USA), supplemented with 10% FBS (Greiner Bio‐One, Alphen aan den Rijn, the Netherlands), 1 mM sodium pyruvate (Thermo Fisher Scientific, California, USA), and 1% v/v MEM non‐essential amino acids solution 100× (Capricorn Scientific GmbH, Ebsdorfergrund, Germany)). Human induced pluripotent stem cells (hiPSCs) (American Type Culture Collection (ATCC) #ACS‐1026, lot no. 0238, Virginia, USA; RRID:CVCL_0A05) were cultured on Geltrex lactose dehydrogenase elevating virus‐free, human embryonic stem cell‐qualified, Reduced Growth Factor Basement Membrane Matrix‐coated wells (#A1413302, Thermo Fisher Scientific, California, USA). hiPSCs were kept in Essential 8 Flex medium (E8 medium) (#A2858501, Thermo Fisher Scientific, California, USA), refreshed every 2 days. After passage, hiPSCs were kept in Essential 8 medium supplemented with 2 μM thiazovivin (#SML1045, Sigma‐Aldrich, Missouri, USA) and replaced with plain E8 medium. All cells were kept in a humidified 37°C incubator with 5% CO_2_ unless stated otherwise.

### 2.4 T‐REx HeLa Cell Line Generation

1.8 × 10^6^ T‐REx HeLa cells (Invitrogen, Massachusetts, USA) were seeded into a 10‐cm petri dish and 6 h later, the cells were co‐transfected with 1 μg pOG44 plasmid (Invitrogen, Massachusetts, USA; K650001) and 1 μg of either empty pcDNA5/FRT/TO‐eGFP (mock), pcDNA5/FRT/TO‐eGFP *RRAGD* WT, or p.(Ser76Leu) plasmid, totaling to 2 μg DNA. As a negative control, cells were transfected with 2 μg of only pOG44 plasmid. For transfection, 2 μL Lipofectamine 2000 (Invitrogen, Massachusetts, USA) was used per 1 μg of DNA. Eighteen hours later, selection was started using 3 μg/mL blasticidin (Sigma‐Aldrich, Missouri, USA) and 100 μg/mL Hygromycin B (Thermo Fisher Scientific, California, USA). T‐REx HeLa cell culture medium supplemented with antibiotics is now termed selection medium. The selection medium was replaced every 2 days until cell sorting.

Nine days after transfection and 24 h before sorting, expression of GFP or fusion GFP‐RagD was induced in the transfected cells by adding 1 μg/mL tetracycline to the selection medium. The following day, the cells were sorted through a fluorescence‐activated cell sorting (FACS) machine by the flow cytometry facility at Radboud University Medical Center, the Netherlands. In short, cells were disassociated from the petri dishes using trypsin–EDTA solution (0.05% trypsin, 0.02% EDTA) and resuspended in PBS + 2% (w/v) EDTA. Individual GFP‐positive cells were sorted into a single 96‐well plate using a 100 μm nozzle of the Cytek Aurora CS System (Cytek, the Netherlands). After 10 days, grown clones from each cell line were expanded and genotyped using the subcloning primers. For each mock, *RRAGD* WT, and p.(Ser76Leu) cell line, one clone was selected and used for subsequent experiments.

### 2.5 T‐REx HeLa Cells Transfection and Treatment

For immunoblotting and RT‐qPCR experiments, 200 000 T‐REx HeLa cells were seeded into a 6‐well plate. The next day, expression of GFP or GFP‐RagD was induced using 1 μg/mL tetracycline. For immunocytochemistry experiments, 300 000 cells were seeded into a 6‐well plate. After 6 h, the cells were transfected with 0.5 μg of pcDNA3.1‐WT‐TFEB‐MYC using FuGENE HD Transfection Reagent (Promega, Shanghai, China) at a 1:2 DNA:FuGENE ratio. The next day, these transfected T‐REx HeLa cells were collected, and 20 000 cells were re‐seeded on poly‐L‐lysine‐coated 10 mm coverslips in 24‐well plates. After 24 h, 1 μg/mL tetracycline was added to the culture medium to induce the expression of GFP or GFP‐RagD. All cells were harvested 24 h after tetracycline addition.

For drug treatment, 24 h after tetracycline induction, T‐REx HeLa cells were treated with 100 nM rapamycin (#SC‐3504A, Santa Cruz Biotechnology, Texas, USA) or 250 nM Torin1 (#4247, Tocris, Abingdon, UK) for 1 h. As a control, 0.01% solvent DMSO (Sigma‐Aldrich, Missouri, USA) was used.

### 2.6 Genome Targeting With CRISPR‐Cas9

gRNAs were designed using CRISPOR web tool (RRID:SCR_015935) with no changes to the default parameters [28]. Six gRNAs were selected (Figure S2), subcloned into the PX458 plasmid, and confirmed by sequencing using the human U6 promoter primer (Table S1). Next, a T7 endonuclease assay was performed to assess the cutting efficiency of each gRNA [29]. To do this, HEK293 cells were transfected with 1 μg of gRNA in a 12‐well plate using 9 μg of 1 mg/mL polyethylenimine (PEI, Polysciences, Pennsylvania, USA). After 48 h, gDNA of HEK293 cells were isolated using proteinase K at 55°C for 2 h. Next, *RRAGD* was amplified using PCR and 1 μL T7 Endonuclease I (New England Biolabs, Massachusetts, USA) was added to the PCR product and incubated for 30 min at 37°C. Digested products were run on agarose gel for analysis. Based on the cutting efficiency and proximity to the region of interest, the most efficient gRNA (i.e., gRNA #5) was selected (Table S1).

To introduce the *RRAGD* c.227C>T mutation, hiPSCs were pre‐treated with thiazovivin for 1 h at 37°C. Then, 3 × 10^6^ hiPSCs suspension was collected and resuspended in 100 μL prewarmed nucleofection mix (Lonza Human Stem Cell Nucleofector Kit 1 #VPH‐5012, Lonza, Oss, the Netherlands) supplemented with 5 μg PX458 containing gRNA #5 and 1 μg of each WT and p.(Ser76Leu) double‐stranded oligonucleotides (gBlocks Gene Fragments, IDT, Iowa, USA). These hiPSCs were then transferred to an electroporation cuvette and nucleofected using the A‐203 program of the Nucleofector device (Lonza, Oss, the Netherlands). Nucleofected hiPSCs were supplemented with warm E8 medium containing 2 μM thiazovivin and transferred to a Geltrex‐coated 6‐well plate. Forty‐eight hours post‐nucleofection, hiPSCs were disassociated using Versene Solution (#15040066, Thermo Fisher Scientific, California, USA). Single cells were then sorted into 96‐well plates based on GFP signal using FACSJazz (BD Biosciences, Drachten, the Netherlands). The 96‐well plates were coated with irradiated mouse embryonic fibroblasts (Thermo Fisher Scientific, California, USA). After sorting, the 96‐well plates were centrifuged for 1 min at 200× *g* and incubated at 37°C.

Fourteen days after the sorting, hiPSC clones were collected using Versene Solution and passaged into two wells of a 96‐well plate. With one well, genotyping was performed to check for the mutation of interest. In short, the gDNA of the clones was isolated using an ethanol‐based precipitation method. Next, a genomic region containing the mutation of interest was amplified with PCR with *RRAGD* genotyping primers (Table S1). Subsequently, Sanger sequencing was done to confirm the presence of the mutation. Maintenance of the clones was carried out using the other well.

After obtaining a hiPSCs clone with mutation of interest and its isogenic controls, the top three off‐target sites predicted by the CRISPOR tool were amplified by PCR and sent for Sanger sequencing (Table S1).

### 2.7 Bulk Karyo‐Sequencing

Approximately 1000 hiPSCs were collected and spun down as pellets. The cell pellets were digested in 5 μL of 2 μg Proteinase K (New England Biolabs, Massachusetts, USA) in 1× CutSmart Buffer (New England Biolabs, Massachusetts, USA) for 2 h at 55°C followed by 10 min at 80°C. To obtain DNA fragments, DNA was digested using 10 μL of 10 U NLAIII (New England Biolabs, Massachusetts, USA) in 1× CutSmart Buffer for 2 h at 37°C followed by 20 min at 80°C. The DNA fragments were ligated to adapters by adding 20 μL or 800 U T4 DNA Ligase (New England Biolabs, Massachusetts, USA), 1 mM ATP (Thermo Fisher Scientific), and 50 nM adapter in 1× T4 DNA Ligase Buffer (New England Biolabs, Massachusetts, USA) and incubated at 16°C overnight. The library preparation, sequencing, and analysis were performed as described previously [30].

### 2.8 Whole Genome Sequencing

Whole genome sequencing (WGS) was performed at the Department of Human Genetics of the Radboud University Medical Center, the Netherlands. Automated DNA isolation was performed using the Nimbus Presto (Hamilton, Maine, USA) using the Kingfisher Presto unit (Thermo Fisher Scientific, California, USA) and Mag‐Bind Blood & Tissue DNA HDQ 96 kit (Omega Biotek, Georgia, USA). To do so, five million hiPSCs were collected and spun down as pellets. Before loading into the Nimbus, the pellets were resuspended in PBS. Libraries were prepared using the NEBNext Ultra II DNA PCR‐free Library Prep Kit (New England Biolabs, Massachusetts, USA) for Illumina sequencing. DNA was sheared into fragments of approximately 450 bp using the Covaris R230 system (Covaris, Massachusetts, USA). WGS libraries were sequenced (paired‐end, 2× 151 bp) on the Illumina NovaSeq X plus (Illumina, California, USA) using a 25B flow cell. Data was analyzed using Illumina DRAGEN (v4.3.13‐3), using GRCh38 as reference and Q30 as a base quality score. Variant annotation was done using an in‐house developed pipeline. For analysis, variants were filtered based on the following criteria: non‐synonymous, located in exon or splice regions, > 10 variation.reads.

### 2.9 Cell Growth Measurement

2500 hiPSCs were seeded on a 96‐well plate coated with Geltrex. The plates were then placed back in a 37°C incubator to allow the cells to attach. Four hours later, they were transferred to the IncuCyte ZOOM live cell imaging system (Essen BioScience, Michigan, USA). Cell growth was recorded every 12 h for a total duration of 96 h. The E8 culture medium was refreshed every 2 days. At the end of the experiment, a mask generated in the system program was used to select cell clusters and quantify the cell growth.

### 2.10 Cardiomyocyte Differentiation

At the start of the differentiation, hiPSCs were maintained and grown until 80%–90% confluency (Day 0). Then, the medium was aspirated, and the cells were rinsed once with HBSS (Thermo Fisher Scientific, California, USA). Differentiation medium (RPMI 1640 with GlutaMAX Supplement with HEPES (#72400‐021, Thermo Fisher Scientific, California, USA), supplemented with 0.5 mg/mL human recombinant albumin (#A9731, Sigma‐Aldrich, Missouri, USA), 0.2 mg/mL L‐Ascorbic Acid 2‐Phosphate (#681671, Sigma‐Aldrich, Missouri, USA)) was added with the addition of 4 μm CHIR99021 (#361559, Sigma‐Aldrich, Missouri, USA). On day 2, the medium was aspirated, and cells were rinsed once with HBSS. Afterward, the differentiation medium was replaced with fresh differentiation medium supplemented with 5 μM IWP‐2 (#681671, Sigma‐Aldrich, Missouri, USA). On days 4 and 6, the medium was again refreshed, but with plain differentiation medium. From day 8, or when the cells started beating, the differentiation medium was replaced every 3–4 days with a cardio culture medium. At day 14, hiPSC‐derived cardiomyocytes (hiPSC‐CMs) were cultured in cardio culture medium (RPMI‐1640 Medium with GlutaMAX Supplement with HEPES supplemented with B‐27 Supplement 50×‐serum free (#17504001, Thermo Fisher Scientific, California, USA)). These hiPSC‐CMs were expanded 9–10× the surface area on Geltrex‐coated wells and flasks for further applications. One and 4 days after expansion, the culture medium was refreshed. After 4 days, the medium was changed once a week.

### 2.11 Cardiomyocyte Culture

The hiPSC‐CMs used in this study are aged 30 or 80 days post‐differentiation. Ten days before the start of the experiment, the hiPSC‐CMs were dissociated using 10× TrypLE Select Enzyme without phenol red (#A1217703, Thermo Fisher Scientific, California, USA) for a maximum of 45 min at 37°C and replated onto Geltrex‐coated surfaces.

For immunoblotting, 250 000 hiPSC‐CMs were replated onto a 6‐well plate. For immunocytochemistry experiments, cells were replated onto a 10 mm coverslip pre‐coated with Geltrex. For electrophysiology experiments, 25 000 cells were replated onto a Geltrex‐coated 10 mm coverslip.

### 2.12 Cardiomyocyte Purity

Approximately 1 × 10^6^ hiPSC‐CMs were disassociated and spun down (300× *g* for 5 min). The resulting pellets were rinsed once with HBSS and fixed using 1 mL ice‐cold 70% EtOH. Next, cells were centrifuged for 4 min at 300× *g*, and the supernatant was removed. Subsequently, cells were resuspended in blocking buffer (PBS supplemented with 5% (v/v) FBS, 1% (w/v) bovine serum albumin, and 0.5% (v/v) Triton X‐100) and incubated for 10 min on ice. Afterward, permeabilized cells were centrifuged for 4 min at 300× *g*, and the supernatant was removed. The cell pellet was then incubated in a blocking buffer supplemented with anti‐cTnT or anti‐ACTN2 for 1 h at 4°C. After 1 h, 500 μL blocking buffer was added, and cells were centrifuged for 4 min at 300× *g*. The supernatant was aspirated, and cells were washed with 500 μL blocking buffer before being spun down again. After this, cells were resuspended in 100 μL blocking buffer supplemented with Alexa Fluor 488 anti‐rabbit or ‐mouse. Following a 30‐min incubation at RT, cells were washed 2× with 500 μL blocking buffer, each followed by a centrifugation step for 4 min at 300× *g*. Finally, the supernatant was aspirated, the cells were resuspended in 1 mL PBS, and analyzed using FACSLyric 12 color (BD Biosciences, Drachten, the Netherlands).

### 2.13 Electrophysiology

hiPSC‐CMs on coverslips were incubated with either Powerload and FluoVolt (1:1000, Thermo Fisher Scientific, California, USA) or Fluo‐4‐AM (1:1000, Thermo Fisher Scientific, California, USA) in cardio culture medium for 20 min at 37°C. Next, the coverslips were placed in FluoroBrite (Thermo Fisher Scientific, California, USA) and kept at 37°C. Cell clusters were imaged using a custom‐built microscope (Cairn Research, Kent, UK) and were paced with field stimulation at 1 Hz using an isolated stimulator (Hugo Sachs Stimulator CS). Fluorescent signals were recorded using a 10× water immersion objective (Olympus UMPlanFl 10×/0.30 W). Dye excitation was done using an excitation filter of 482/35 nm. Fluorescent signals were captured through a long‐pass emission filter (514 nm) by a high‐speed camera (Andor Zyla 5.5CL3, Oxford Instruments, Abingdon, UK). Data analysis was performed in FIJI and a custom MATLAB script (RRID:SCR_001622; DOI 10.17605/OSF.IO/86UFE).

### 2.14 RNA Isolation and RT‐qPCR

From T‐REx HeLa cells, total RNA was extracted using the NucleoSpin RNA Plus kit (Macherey‐Nagel, Dueren, Germany) per the manufacturer's protocol. From hiPSC‐CMs, Trizol Reagent (Invitrogen, Bleiswijk, the Netherlands) was used according to the manufacturer's protocol. Next, 1 μg of isolated RNA was treated with DNase (Promega, Wisconsin, USA) and reverse transcribed with Moloney Murine Leukemia Virus Reverse Transcriptase (Invitrogen, Bleiswijk, the Netherlands).

Gene expression was quantified using SYBR Green (Bio‐Rad, California, USA) on a CFX96 Real‐Time PCR Detection System (Bio‐Rad, California, USA). The 2^−ΔΔCt^ method was used to calculate relative gene expression, normalized to the geometric mean of the housekeeping genes. For HeLa T‐REx cells, the housekeeping genes used were *GAPDH* and *RPLP0*. Values are presented as fold changes against the control group. The genes were selected based on the highest differential expression and their association with cardiomyopathies. All primer sequences used are listed in Table S1.

### 2.15 Bulk RNA‐Seq

RNA‐Seq libraries were prepared from total RNA using the KAPA RNA HyperPrep Kit with RiboErase (KAPA Biosystems, Massachusetts, USA). Oligo hybridization, rRNA depletion and cleanup, DNase digestion and cleanup, and RNA elution were performed according to the manufacturer's protocol. Next, fragmentation and priming were performed at 94°C for 6 min. Synthesis of the first‐ and second‐strands and A‐tailing was performed according to the protocol. After that, the adaptor was ligated using NextFlex DNA barcodes (1.5 mM stock; Bio Scientific, Texas, USA), and the first and second post‐ligation cleanup was performed according to the protocol. To amplify the library, 11 PCR cycles were performed, and the library was further cleaned up using a 0.8× followed by a 1.0× bead‐based cleanup. The size of the library was determined using the High Sensitivity DNA bioanalyzer kit, and the library concentration was measured using the dsDNA High Sensitivity Assay (DeNovix, Delaware, USA). Finally, paired‐end sequencing reads of 50 bp were generated using an Illumina NextSeq 2000.

### 2.16 RNA‐Seq Data Analysis

RNA‐Seq data were analyzed using the seq2science pipeline (https://vanheeringen‐lab.github.io/seq2science/content/workflows/rna_seq.html). In short, reads of all genes were aligned to the human GRCh38.p14 reference genome. Next, reads were filtered using SAMtools (RRID:SCR_002105). Quality score < 20 and PCR duplicates were removed [31]. Reads per gene were counted with the htseq‐count script from the Hisat2 software suite (RRID:SCR_015530) using the GTF file corresponding to the transcript assembly. Read counts were further analyzed with DESeq2 (RRID:SCR_002285) [32]. Bulk RNA‐Seq data have been deposited in the National Center for Biotechnology Information Gene Expression Omnibus (GEO) database (Accession No. GSE323990).

### 2.17 Amino Acids Stimulation

Amino acids (AA) stimulation was performed 24 h after tetracycline induction in T‐REx HeLa cells. Homemade nutrient‐rich (+AA) and starvation (−AA) media were made following the culture medium manufacturer's formulation (see the cell culture section for the media). The −AA medium was prepared by omitting the addition of any AA to the medium. To both +AA and −AA media, 1% (v/v) 100× MEM vitamin solution (Thermo Fisher Scientific, USA) was added. The media were then pH‐adjusted with 2 M HCl and sterile filtered through a 0.22 μm filter device. Before use, treatment media were supplemented with 10% (v/v) dialysed FBS (Thermo Fisher Scientific, USA) and 1 mM sodium pyruvate. Specifically to +AA medium, 4 mM L‐glutamine (Thermo Fisher Scientific, USA) and 1% (v/v) MEM non‐essential amino acids solution 100× (Capricon Scientific, Germany) were added. Prior to the addition of +AA or −AA medium, the cells were washed with PBS. Subsequently, the PBS was replaced with +AA or −AA medium for 1 h and incubated at 37°C.

For hiPSC‐CMs, homemade +AA and −AA media were prepared according to the RPMI 1640 with GlutaMAX Supplement with HEPES recipe without AA. Only to +AA medium, 50× RPMI 1640 Amino Acids Solution (Sigma‐Aldrich, Missouri, USA) and 2 mM GlutaMAX (Sigma‐Aldrich, Missouri, USA) were added. The media were then pH‐adjusted with 2 M HCl and sterile filtered through a 0.22 μm filter device. Before use, both +AA and −AA media were supplemented with 50× B‐27 Supplement serum free. Prior to treatment, hiPSC‐CMs were rinsed once with HBSS. Afterward, HBSS was removed, and +AA or −AA medium was added to the cells and incubated for 1 h at 37°C.

### 2.18 Immunoblotting

Cells were washed once in the wells with ice‐cold PBS prior to lysis in Triton lysis buffer (50 mM Tris–HCl pH 7.5, 1 mM EGTA, 1 mM EDTA, 1% v/v Triton X‐100, 10 mM Na‐glycerophosphate, 50 mM NaF, 10 mM Na‐pyrophosphate, 270 mM sucrose, and 150 mM NaCl) supplemented with phosphatase inhibitor (1 mM Na‐orthovanadate) and protease inhibitors (1 mM PMSF, 1 μM aprotinin, 0.01 mM leupeptin, 1.4 μM pepstatin) by scraping on ice. Next, samples were centrifuged at 4°C at 13 000 rpm for 10 min, and the supernatant was collected. The protein concentration was measured using the Pierce BCA protein assay kit (Thermo Scientific, California, USA) and 1 μg/μL protein samples were prepared in Laemmli + DTT loading buffer. Finally, samples were denatured at 95°C for 5 min and stored at −20°C until use.

Fifteen μg of proteins were loaded onto a 12% polyacrylamide gel and subjected to SDS‐PAGE. After SDS‐PAGE, the proteins were transferred to polyvinylidene fluoride (PVDF) membranes at 100 V for 120 min. Following the transfer, similar protein loading was confirmed using Ponceau S staining (Thermo Fisher Scientific, California, USA). The membranes were then blocked in 5% (w/v) non‐fat dry milk in Tris‐buffered saline (TBS) containing 0.1% (v/v) Tween‐20 (TBS‐T; Sigma‐Aldrich, Missouri, USA). After 1 h of blocking, the membranes were incubated in primary antibody diluted in 1% (w/v) non‐fat dry milk in TBS‐T at 4°C. The following day, the membranes were washed three times in TBS‐T and incubated with secondary antibody in 1% milk in TBS‐T at room temperature for 1 h. Afterward, the membranes were washed thrice in TBS‐T and once in TBS. To visualize the protein of interest, membranes were incubated with SuperSignal West Pico Chemiluminescent Substrate (Thermo Fisher Scientific, California, USA) or SuperSignal West Femto Maximum Sensitivity Substrate (Thermo Fisher Scientific, California, USA) and then imaged using the ImageQuant LAS 4000 (General Electric Healthcare Life Sciences, Uppsala, Sweden).

### 2.19 Immunocytochemistry

Cells on coverslips were fixated with 4% (v/v) paraformaldehyde (Sigma‐Aldrich, Missouri, USA) in PBS for 10 min at RT. Subsequently, the cells were permeabilized for 10 min with PBS containing 0.3% (v/v) Triton X‐100 and 0.1% (w/v) bovine serum albumin. Followed by incubation in 50 mM NH_4_Cl in PBS for 10 min. After two rinses with PBS, the coverslips were blocked in goat serum dilution buffer (GSDB) containing 16% (v/v) goat serum and 0.3% Triton X‐100 in PBS for 30 min and incubated in primary antibodies diluted in GSDB overnight at 4°C. The next day, the coverslips were washed thrice with PBS and incubated with secondary antibodies diluted in GSDB for 45 min at RT. The samples were then again washed thrice with PBS. During the second wash, DAPI was added to the PBS to a final concentration of 300 nM. Finally, samples were mounted with Fluoromount‐G (Southern Biotech, Alabama, USA). Images were taken with a SP8 confocal microscope equipped with a 63× water‐based objective (NA 1.20) (Leica Microsystems, Amsterdam, the Netherlands), or, specifically for the RRAGD‐LAMP1 co‐localization, a LSM900 confocal microscope with Airyscan 2 equipped with a 63× oil‐based airyscan objective (NA 1.40) (Carl Zeiss BV, Breda, the Netherlands). In all cases, system‐specific excitation wavelengths and emission detection ranges were used that were optimal for the used fluorophores with minimal spillover between channels. Airyscanning post‐processing was performed according to the manufacturer's protocol (Carl Zeiss BV) on RRAGD‐LAMP1 co‐localization images to obtain maximal spatial resolution.

To quantify TFEB nuclear translocation, automated scripts were created in ImageJ2 version 2.14.0 (RRID:SCR_003070). For T‐REx HeLa cells, using the TFEB channel, TFEB‐transfected cells were manually selected using the free‐shape function. The DAPI channel was used to determine the location and outline of the nuclei. Next, a ratio of the TFEB signal in the nuclei over the total TFEB signal in cells per image was calculated, thus depicting the TFEB translocation to the nuclei. For hiPSC‐CMs, z‐stacks consisting of 5 to 7 *z*‐plane images per stack were acquired. Next, a similar automated script was applied to calculate DAPI intensity, the TFEB signal in the nuclei, and in total cells, but without manually selecting cells as endogenous TFEB expression was analyzed. Afterward, within each stack, the *z*‐plane image with the brightest DAPI signal (i.e., the middle plane of the nuclei) was selected and used for quantification. In each experiment, 10 images from 10 independent fields were obtained per condition. In the end, the average of the 10 images per genotype was calculated.

### 2.20 Statistics

All results are depicted as individual values and mean ± SEM from independent experiments, meaning different passages/differentiations of cells as well as separated experimental steps. The statistical test used in each experiment is indicated in each figure legend. Statistical significance was described at *p* < 0.05. All graphs and statistical tests were run in GraphPad Prism version 10.4.1 (532) for macOS (GraphPad Software, Massachusetts, USA; RRID:SCR_021836).

## 3 Results

### 3.1 *RRAGD* p.(Ser76Leu) Variant Impairs mTORC1 Signaling Response to Amino Acids

**FIGURE 1:**
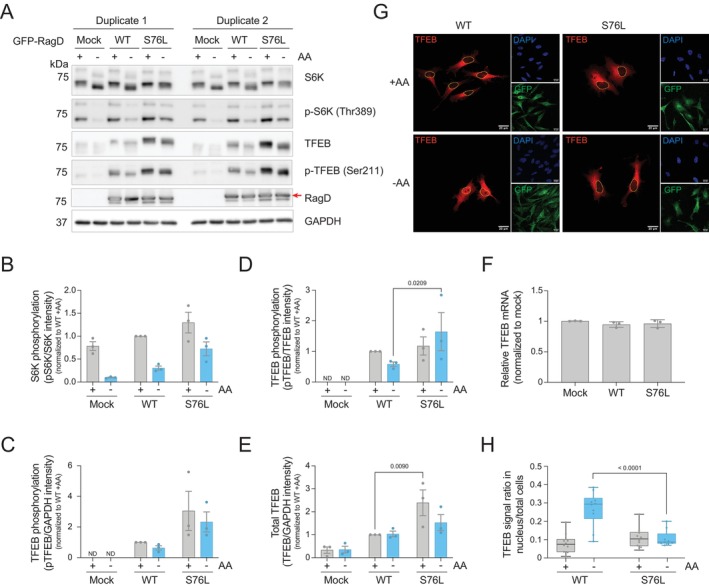
*RRAGD* p.(Ser76Leu) variant constitutively activate mTORC1 in T‐REx HeLa cells. (A–E) T‐REx HeLa cells stably overexpressing GFP (mock), GFP‐*RRAGD* WT (WT), and GFP‐*RRAGD* p.(Ser76Leu) (S76L) were exposed amino acids‐containing medium (+AA, gray bars) or amino acids‐deprived medium (−AA, blue bars). (A) Representative immunoblots of S6K, p‐S6K, TFEB, p‐TFEB, GFP, and GAPDH after amino acids stimulation. (B–E) Quantification of (B) phosphorylated S6K/total S6K ratio, (C) phosphorylated TFEB corrected to GAPDH, (D) phosphorylated TFEB/total TFEB ratio, and (E) total TFEB corrected to GAPDH (mean ± SEM from three independent experiments, normalized to the WT + AA condition). (F) mRNA levels of TFEB in mock, *RRAGD*‐WT, and *RRAGD*‐p.(Ser76Leu) T‐REx HeLa cell lines. (G, H) Mock, *RRAGD*‐WT, and *RRAGD*‐p.(Ser76Leu) T‐REx HeLa cell lines transiently transfected with TFEB and exposed to amino acids stimulation. (G) Representative images of TFEB staining (red), GFP‐RagD (green), and DAPI (blue). The nuclei of cells that were transfected with TFEB are outlined in yellow. Scale bars indicate 20 μm. (H) Quantification of TFEB signal in the nucleus/in the total cells. Boxes indicate the 25th to 75th quartile; middle lines indicate the median, and whiskers extend from maximum to minimum one independent experiment with images from 10 independent fields per condition. For statistical analyses, the following tests were done. (B–E) Two‐way ANOVA comparison between mock or p.(Ser76Leu) cells and WT cells within treatment (i.e., +AA and −AA), followed by Dunnett's test. (G) One‐way ANOVA and Tukey's test. The normality of the distribution was tested using a Q‐Q plot. (H) Two‐way ANOVA, where the effects of genotypes within each treatment were compared, followed by Šídák's test. The normality of the distribution was tested using a Q‐Q plot. ND, not detectable.

To study the effect of the *RRAGD* p.(Ser76Leu) variant on amino‐acid‐induced mTORC1 signaling, T‐REx HeLa cell lines overexpressing GFP (mock) or GFP‐*RRAGD* WT or p.(Ser76Leu) were stimulated with amino acids. S6K phosphorylation was not significantly different between *RRAGD* WT and p.(Ser76Leu) cells (Figure 1A,B), although a significant difference was seen between treatment within the genotypes (two‐way ANOVA followed by Šídák's test, mock: +AA 0.78 ± 0.09 vs. −AA 0.09 ± 0.01, *p* = 0.0047; WT: +AA 1.00 ± 0.00 vs. −AA 0.30 ± 0.05, *p* = 0.0042; S76L: +AA 1.29 ± 0.23 vs. −AA 0.73 ± 0.15, *p* = 0.0161). TFEB phosphorylation, when corrected to GAPDH, was also not significantly different between *RRAGD* p.(Ser76Leu) cells and *RRAGD* WT cells (Figure 1C). In amino acids deprived conditions (−AA), TFEB phosphorylation was significantly higher in *RRAGD* p.(Ser76Leu) cells compared to *RRAGD* WT cells (p‐TFEB/TFEB: 1.65 ± 0.62 vs. 0.58 ± 0.08; Figure 1D). Of note, overexpression of *RRAGD* p.(Ser76Leu) resulted in significantly higher TFEB expression than WT cells (Figure 1E). Endogenous TFEB mRNA expression, however, was not different between all three cell lines (Figure 1F). To examine the effects of *RRAGD* p.(Ser76Leu) on nuclear translocation of TFEB, cells were stained for TFEB after exposure to +AA or −AA conditions. *RRAGD* p.(Ser76Leu) cells had a significantly lower relative nuclear TFEB signal compared to *RRAGD* WT cells under −AA conditions (0.11 ± 0.02 vs. 0.27 ± 0.03; Figure 1G,H).

**FIGURE 2:**
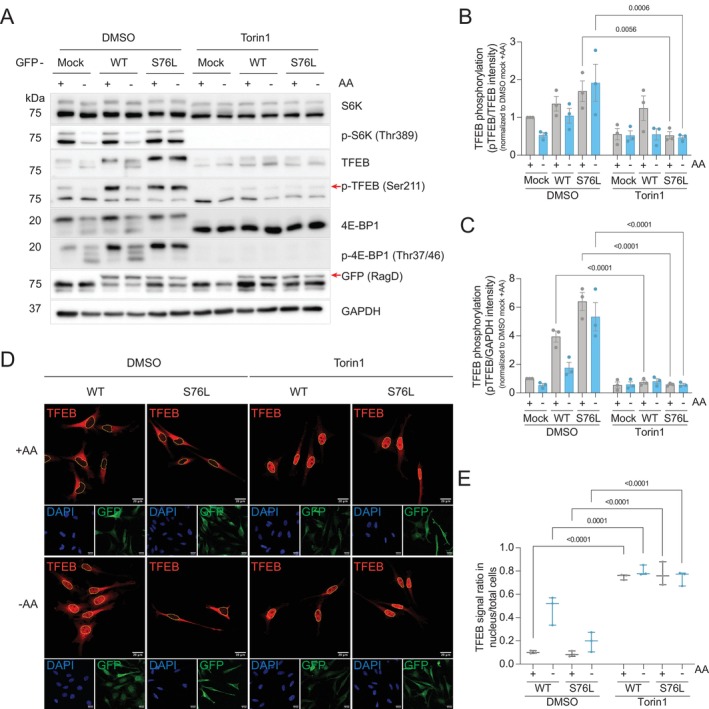
mTOR inhibition by Torin1 restored mTORC1 signaling in *RRAGD* p.(Ser76Leu) T‐REx HeLa cells. (A–E) T‐REx HeLa cells stably overexpressing GFP (mock), GFP‐*RRAGD* WT (WT), and GFP‐*RRAGD* p.(Ser76Leu) (S76L) were exposed to amino acids‐containing medium (+AA, gray bars) or amino acids‐deprived medium (−AA, blue bars) and Torin1 or DMSO. (A) Representative immunoblots of S6K, p‐S6K, TFEB, p‐TFEB, 4E‐BP1, p‐4E‐BP1, GFP, and GAPDH following treatment mentioned above. (B, C) Quantification of (B) phosphorylated TFEB/total TFEB ratio and (C) phosphorylated TFEB/GAPDH ratio (mean ± SEM from three independent experiments, normalized to the DMSO mock +AA condition). (D, E) Mock, *RRAGD*‐WT, and *RRAGD*‐p.(Ser76Leu) T‐REx HeLa cell lines transiently transfected with TFEB and exposed to amino acids stimulation and Torin1 or DMSO. (D) Representative immunofluorescence images of TFEB staining (red), GFP‐RagD (green), and DAPI (blue). TFEB‐positive nuclei are outlined in yellow. Scale bars indicate 20 μm. (E) Quantification of TFEB signal in the nucleus/in the total cells. Vertical lines indicate the 25th to 75th quartile, horizontal lines indicate the median, and whiskers extend from maximum to minimum from three independent experiments with images from 10 independent fields per condition. For statistical analyses, Two‐way ANOVA was performed in which a comparison between DMSO and Torin1 treatment within the genotypes and +AA or −AA was made, followed by Šídák's test. The normality of the distribution was tested using a Q‐Q plot.

To assess whether the constant phosphorylation of S6K, 4E‐BP1, and TFEB depends on mTORC1 activity, the cells were treated with the pharmacological mTORC1 inhibitors Torin1 (Figure 2) and rapamycin (Figure S1). In line with S6K phosphorylation (Figure 1A,B), 4E‐BP1 phosphorylation seemed to persist in −AA + DMSO conditions in *RRAGD* p.(Ser76Leu) cells, unlike in mock and *RRAGD* WT cells (Figure 2A). Inhibition of mTOR by Torin1 diminished S6K and 4E‐BP1 phosphorylation, and significantly lowered TFEB phosphorylation in both +AA and −AA conditions in p.(Ser76Leu) cells compared to DMSO treatment (Torin1 vs. DMSO; +AA p‐TFEB/GAPDH: 0.58 ± 0.05 vs. 6.41 ± 0.63; +AA p‐TFEB/TFEB: 0.53 ± 0.09 vs. 1.69 ± 0.27; −AA p‐TFEB/GAPDH: 0.58 ± 0.09 vs. 5.34 ± 0.99; −AA p‐TFEB/TFEB: 0.47 ± 0.05 vs. 1.92 ± 0.49; Figure 2A–C). Moreover, immunofluorescence on TFEB indicated that Torin1 treatment significantly increased the nuclear re‐localization of TFEB compared to DMSO treatment under both +AA and −AA conditions in *RRAGD* p.(Ser76Leu) cells that were transiently transfected with TFEB (Torin1 vs. DMSO; +AA 0.78 ± 0.06 vs. 0.08 ± 0.01; −AA 0.74 ± 0.04 vs. 0.19 ± 0.05; Figure 2D,E). Inhibition of mTORC1 signaling by rapamycin abolished the phosphorylation of S6K and 4E‐BP1 but did not affect phosphorylated TFEB (Figure S1A–C).

### 3.2 Generation of *RRAGD* ^WT^ ^/p.Ser76Leu^ Induced‐Pluripotent Stem Cell‐Derived Cardiomyocytes

**FIGURE 3:**
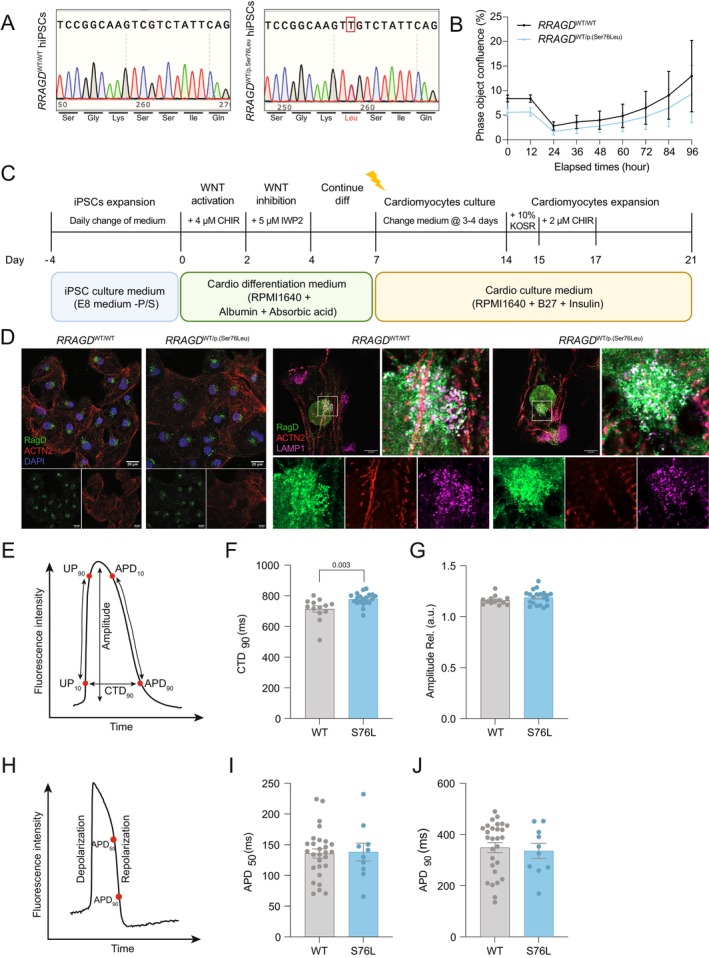
Generation of *RRAGD* ^WT/p.(Ser76Leu)^ human induced pluripotent stem cell‐derived cardiomyocytes (hiPSC‐CMs). (A) Sequencing results of *RRAGD* ^WT/WT^ (left) and *RRAGD* ^WT/p.(Ser76Leu)^ (right) hiPSCs clones. The red box highlights *RRAGD* c.227C>T (p.(Ser76Leu)) mutation. (B) Representative growth curve of *RRAGD* ^WT/WT^ (black line) and *RRAGD* ^WT/p.(Ser76Leu)^ (blue line) hiPSCs (mean ± SD). (C) hiPSC‐CMs differentiation scheme. (D) Representative immunofluorescence image of *RRAGD* ^WT/WT^ and *RRAGD* ^WT/p.(Ser76Leu)^ hiPSC‐CMs stained with RagD (green), sarcomere ACTN2 (red), and DAPI (blue) or LAMP1 (magenta). (E–J) Electrophysiological assays of *RRAGD* ^WT/WT^ (gray bar) and *RRAGD* ^WT/p.(Ser76Leu)^ (blue bar) hiPSC‐CMs. (E) Illustration of Ca^2+^ transient assay parameters depicting the amplitude, 10% and 90% upstroke (UP) and downstroke (APD), and the 90% Ca^2+^ transient duration (CTD). (F) CTD_90_ represents the total duration of the Ca^2+^ transient. (G) Relative amplitude represents the amount of Ca^2+^ in the initial release. Data are mean ± SEM from 13 to 20 cell clusters from one differentiation round. (H) Illustration of action potential parameters depicting 50% and 90% of the downstroke of the fluorescence intensity, also known as action potential duration (APD). (G) 50% and (H) 90% of APD (APD 50 and 90, respectively). Data are mean ± SEM from 10 to 28 cell clusters from one differentiation round. (F, G, I, and J) Two‐tailed unpaired *t*‐test statistical test was performed. (E and H) Adapted from van Ham et al. [33]

To validate the effects of the *RRAGD* p.(Ser76Leu) variant in a more physiologically relevant model, we generated a human induced pluripotent stem cell (hiPSC) model with an endogenous heterozygous expression of the *RRAGD* p.(Ser76Leu) variant (hereafter referred to as *RRAGD* ^WT/p.(Ser76Leu)^), mediated by the CRISPR‐Cas9 system (Figures 3A and S2A). *RRAGD* ^WT/WT^ hiPSCs are isogenic controls of *RRAGD* ^WT/p.(Ser76Leu)^ hiPSCs, obtained from a clone that also underwent the CRISPR‐Cas9 gene editing, but no mutation was introduced. Characterization of the hiPSCs indicated that cell growth of *RRAGD* ^WT/p.Ser76Leu^ and *RRAGD* ^WT/WT^ hiPSCs was not significantly different (Figure 3B). Furthermore, Sanger sequencing of the top three predicted off‐targets revealed no mistargeting by the sgRNA following CRISPR‐Cas9 gene editing (Figure S2B). This was again confirmed via whole genome sequencing (WGS) on the *RRAGD* ^WT/p.Ser76Leu^ and *RRAGD* ^WT/WT^ hiPSCs. Although the WGS identified variations between the *RRAGD* ^WT/WT^ and *RRAGD* ^WT/p.Ser76Leu^, none of the identified genes were involved in mTOR signaling (Table S2). Additional quality controls through immunofluorescence confirmed that both *RRAGD* ^WT/p.Ser76Leu^ and *RRAGD* ^WT/WT^ hiPSCs remain pluripotent, as evidenced by the positive nuclear staining of pluripotent markers SOX‐2, OCT3/4, and NANOG (Figure S2C,D). Finally, karyo‐sequencing of these hiPSC clones demonstrated a normal chromosome copy number (Figure S2E,F).

Next, these hiPSCs were differentiated into cardiomyocytes (hiPSC‐CMs) (Figure 3C). To determine whether the p.(Ser76Leu) variant affects RagD subcellular localization, we performed immunofluorescence, which showed that in both *RRAGD* ^WT/p.Ser76Leu^ and *RRAGD* ^WT/WT^ hiPSC‐CMs, RagD predominantly localized in the cytoplasm but was also found in the nucleus. Especially the perinuclear RagD co‐localized heavily with the lysosomal marker LAMP1 in both genotypes (Figure 3D). We then employed electrophysiological assays (i.e., Ca^2+^ transient and action potential measurements) to functionally characterize *RRAGD* ^WT/p.Ser76Leu^ hiPSC‐CMs. Our findings indicated that in Ca^2+^ transient measurements (Figure 3E), Ca^2+^ transient duration (CTD_90_) was significantly prolonged in *RRAGD* ^WT/p.Ser76Leu^ hiPSC‐CMs compared to *RRAGD* ^WT/WT^ hiPSC‐CMs (mean ± SEM *RRAGD* ^WT/p.Ser76Leu^ vs. *RRAGD* ^WT/WT^ hiPSC‐CMs; CTD_90_: 782 ± 9.1 ms vs. 717.3 ± 20.5 ms; Figure 3F). Relative Ca^2+^ amplitude was similar between the two cell lines (Figure 3G). The action potential duration (APD; Figure 3H), as measured by the APD_50_ and APD_90_, was not significantly different between *RRAGD* ^WT/p.Ser76Leu^ and *RRAGD* ^WT/WT^ hiPSC‐CMs (Figure 3I,J).

### 3.3 Heterozygous *RRAGD* p.(Ser76Leu) Expression Does Not Affect mTORC1 Signaling in 2D‐Cultured hiPSC‐CMs

**FIGURE 4:**
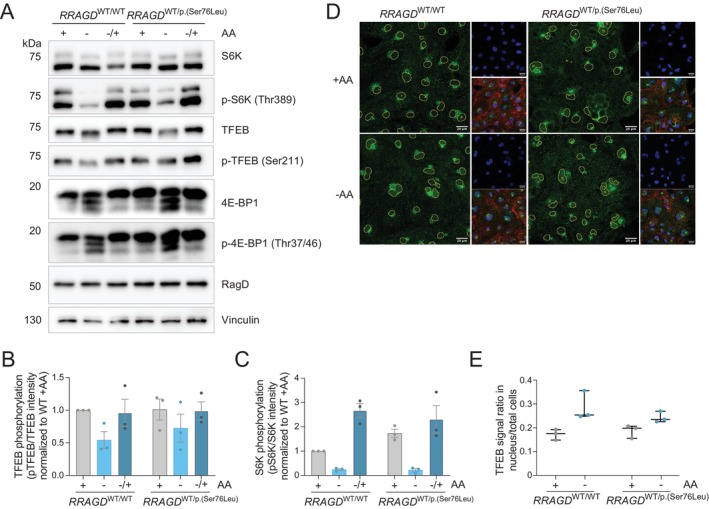
Heterozygous *RRAGD* p.(Ser76Leu) variant did not alter mTORC1 signaling in 2D‐cultured hiPSC‐CMs. (A–E) *RRAGD* ^WT/WT^ and *RRAGD* ^WT/p.(Ser76Leu)^ hiPSC‐CMs were exposed to amino acids stimulation, which are culture medium containing amino acids (+AA), deprived from amino acids (−AA), or −AA followed by re‐supplementation of +AA medium (−/+AA). (A) Immunoblots of S6K, p‐S6K, TFEB, p‐TFEB, 4E‐BP1, p‐4E‐BP1, RagD, and vinculin. (B, C) Quantification of (B) phosphorylated TFEB and (C) S6K (mean ± SEM from three independent hiPSC‐CMs differentiations, normalized to the *RRAGD* ^WT/WT^ +AA condition). Gray bars = +AA, light blue bars = −AA, dark blue bars = −/+AA. (D) Representative immunofluorescence image of *RRAGD* ^WT/WT^ and *RRAGD* ^WT/p.(Ser76Leu)^ hiPSC‐CMs exposed to +AA or −AA and stained for TFEB (green), sarcomeric ACTN2 (red), and DAPI (blue). On the TFEB images, the quantified nuclei area is outlined in yellow. (E) Quantification of TFEB signal ratio in the nucleus over TFEB in total cells. Vertical lines span from the minimum to the maximum point, and horizontal lines depict the median. Gray = *RRAGD* ^WT/WT^ hiPSC‐CMs and blue = *RRAGD* ^WT/p.(Ser76Leu)^ hiPSC‐CMs. (B–E) The statistical test used was two‐way ANOVA, comparing the effects of genotypes within the treatment group (i.e., +AA, −AA, or −/+AA). The normality of the distribution was tested using a Q‐Q plot.

To validate our findings in HeLa cells in 2D‐cultured cardiomyocytes, *RRAGD* ^WT/p.(Ser76Leu)^ and *RRAGD* ^WT/WT^ hiPSC‐CMs were exposed to amino acid starvation, followed by re‐supplementation (i.e., −/+AA). Immunoblotting showed that phosphorylation of S6K, 4E‐BP1, and TFEB was not statistically different in *RRAGD* ^WT/p.(Ser76Leu)^ and *RRAGD* ^WT/WT^ hiPSC‐CMs, also under −/+AA conditions (Figure 4A–C). Accordingly, TFEB subcellular localization was not statistically different in *RRAGD* ^WT/p.(Ser76Leu)^ than that in *RRAGD* ^WT/WT^ hiPSC‐CMs (Figure 4D,E).

### 3.4 Bulk RNA‐Seq Revealed Enriched Pathways in *RRAGD* ^WT^ ^/p.(Ser76Leu)^ hiPSC‐CMs

**FIGURE 5:**
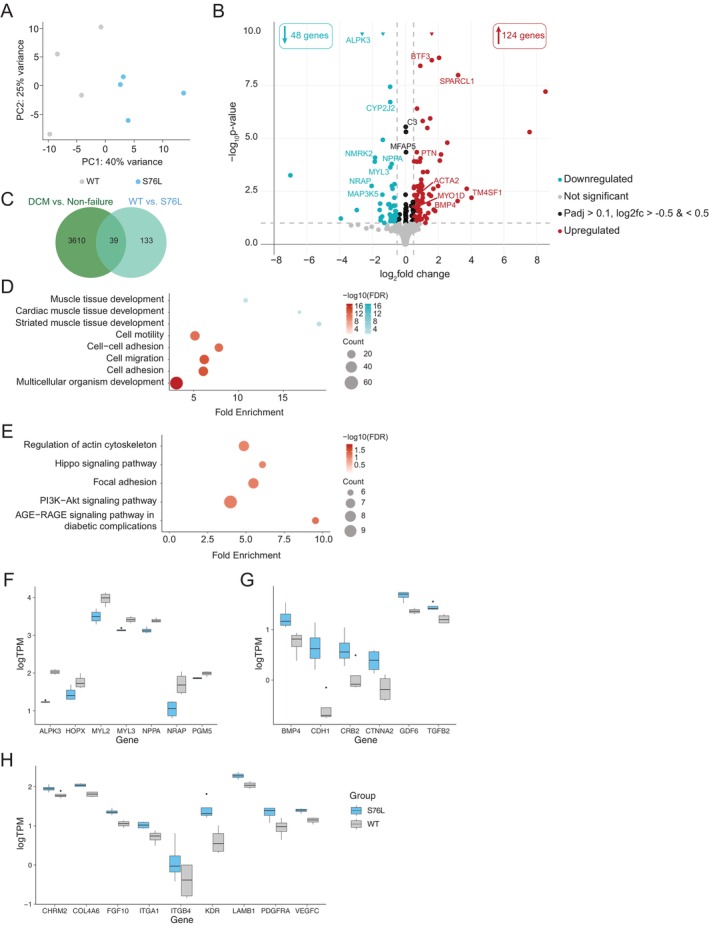
Gene expression profile of 2D‐cultured *RRAGD* ^WT/p.(Ser76Leu)^ hiPSC‐CMs with bulk RNA‐seq. (A) Principal component analysis plot showing the clustering of samples based on genotypes *RRAGD* ^WT/WT^ (WT; gray) and *RRAGD* ^WT/p.(Ser76Leu)^ (S76L; blue) hiPSC‐CMs. Samples are from four independent differentiations. (B) Volcano plot of DESeq2 calculated differentially expressed genes (DEGs; adjusted (adj.) *p* < 0.1) in *RRAGD* ^WT/p.(Ser76Leu)^ hiPSC‐CMs from *RRAGD* ^WT/WT^ hiPSC‐CMs. In total, 172 genes comprising 124 upregulated (red) and 48 downregulated (turquoise) are differentially expressed in *RRAGD* ^WT/p.(Ser76Leu)^ hiPSC‐CMs. Gray dots indicate insignificant DEGs (adj. *p* > 0.1), and black dots indicate fold change of genes > −0.5 and < 0.5. Triangles indicate genes with −log_10_ *p*‐value > 10. Gray dotted lines indicate the cut‐off marks. Genes labeled are significant DEGs overlapping with a previously published RNA‐seq dataset of genes specific to DCM patients (see Table S4) [34]. (C) In the Venn diagram of 172 DEGs with DCM‐specific genes in Sweet et al. [34], 39 genes overlapped. (D, E) Bubble plots showing enriched pathways in *RRAGD* ^WT/p.(Ser76Leu)^ hiPSC‐CMs. (D) Top enriched biological function gene ontology (GO) pathways based on false discovery rate (FDR). Filtered for pathways relevant in cardiomyocytes. (E) Enriched Kyoto Encyclopedia of Genes and Genomes (KEGG) pathways. The intensity of the bubble color is based on −log_10_ FDR; red shows upregulated pathways, and blue shows downregulated pathways. The bubble size indicates the number of DEGs in each pathway (count). (F–H) Box plots of DEGs in (F) muscle developmental pathways, (G) Hippo pathway, and (H) PI3K‐Akt pathway, displaying the log transcripts per million (TPM) of the genes in *RRAGD* ^WT/WT^ (WT; gray) and *RRAGD* ^WT/p.(Ser76Leu)^ (S76L; blue) hiPSC‐CMs samples. The boxes indicate the 1st to 3rd quartile, the middle lines indicate the median, whiskers extend from 1st to 3rd quartile, and black dots indicate the outlier.

To obtain a more complete overview of the cellular consequences of *RRAGD* p.(Ser76Leu) variant in hiPSC‐CMs, bulk RNA‐seq was performed (Figure 5A). In *RRAGD* ^WT/p.(Ser76Leu)^ hiPSC‐CMs, 172 genes were differentially expressed (Table S3). Of these 172 genes, 48 were downregulated, and 124 were upregulated compared to *RRAGD* ^WT/WT^ hiPSC‐CMs (adjusted *p*‐value < 0.1; Figure 5B). Among these differentially expressed genes (DEGs), 39 genes overlapped with a published RNA‐seq dataset from the ventricles of DCM patients [34], which includes key genes (log2 fold change; log2fc to *RRAGD* ^WT/WT^) such as *ALPK3* (−2.64), *BMP4* (1.43), *MYL3* (−0.91), *MYO1D* (0.78), and *NPPA* (−0.83) (Figure 5C and Table S4).

Afterwards, pathway analysis was conducted to investigate the functions of the DEGs. Gene ontology (GO) enrichment analysis indicated that pathways related to muscle tissue development were downregulated, while those linked to cell adhesion and extracellular matrix organization were upregulated in *RRAGD* ^WT/p.(Ser76Leu)^ hiPSC‐CMs (Figures 5D and S3A–C). Further examination using the Kyoto Encyclopedia of Genes and Genomes (KEGG) pathway revealed that pathways such as focal adhesion, actin cytoskeleton regulation, Hippo signaling, and PI3K‐Akt signaling were upregulated in *RRAGD* ^WT/p.(Ser76Leu)^ hiPSC‐CMs (Figure 5E). Upon closer inspection, genes specific to DCM patients [34], including *ALPK3, NPPA, MYL2*, and *MYL3*, were identified within the muscle tissue development pathways (Figure 5F). The dysregulation of these genes was validated via RT‐qPCR, confirming their downregulation in *RRAGD* ^WT/p.(Ser76Leu)^ hiPSC‐CMs (Figure S4A). The validation also showed that although *ACTA2* is upregulated in the RNA‐seq, this could not be confirmed via RT‐qPCR. Furthermore, genes such as *CTNNA2*, *COL4A6*, *ITGA1*, *ITGB4*, and *LAMB1* were present in the upregulated pathways, including cell adhesion, Hippo signaling, and PI3K‐Akt signaling (Figure 5G,H). No mTOR or TFEB‐related pathways or genes were among the significant DEGs in *RRAGD* ^WT/p.(Ser76Leu)^ hiPSC‐CMs when compared to *RRAGD* ^WT/WT^ hiPSC‐CMs.

Because Hippo and PI3K‐Akt signaling activities were upregulated in *RRAGD* ^WT/p.(Ser76Leu)^ hiPSC‐CMs, we examined the phosphorylation of TAZ and Akt as a measure of pathway activation through immunoblotting (Figure S4B). We showed that Akt phosphorylation at the Thr‐308 site was significantly lowered in *RRAGD* ^WT/p.(Ser76Leu)^ hiPSC‐CMs while TAZ phosphorylation at the Ser‐89 site was unchanged (Figure S4C,D).

## 4 Discussion

Pathogenic variants in *RRAGD* cause DCM. Here, we demonstrate that the cardiomyocytes carrying the *RRAGD* p.(Ser76Leu) variant display signs of dedifferentiation and malfunction. These conclusions are based on electrophysiological analysis demonstrating a longer Ca^2+^ transient duration, downregulation of pathways related to muscle development, and upregulation of pathways linked to actin cytoskeleton, extracellular matrix organization, and cell–cell adhesion. We propose that these changes can be attributed to increased canonical and non‐canonical mTORC1 signaling. Taken together, these results provide evidence for cardiomyocyte dysfunction in ADKH‐*RRAGD*.

DCM is a genetically and phenotypically heterogeneous disease with over 50 known causative genes. The mechanisms leading to the manifestation of DCM also differ widely depending on the underlying genetic cause. For example, the most common cause of DCM is genetic variations in genes encoding the sarcomeric components such as titin, myosin, and troponin that lead to cardiomyocyte‐impaired force generation, while variants in genes encoding desmosomal proteins, which are responsible for cell–cell adhesion, are also found in a DCM cohort [35, 36]. Additionally, variants in genes that lead to cytoskeleton disorganization, Ca^2+^ cycling abnormalities, and ion channel dysfunctions have also been associated with DCM [35]. The diversity highlights the need for a thorough understanding of each DCM form and, ultimately, a precision medicine approach to manage it. To understand how the *RRAGD* p.(Ser76Leu) variant influences cardiomyocyte gene expression, we utilized bulk RNA‐seq. Among the DEGs, genes associated with muscle development pathways were downregulated, while genes involved in actin cytoskeleton, extracellular matrix, and cell–cell adhesion pathways were upregulated in *RRAGD* ^WT/p.Ser76Leu^ hiPSC‐CMs. Previous transcriptomic studies with DCM subjects have identified dysregulated mitochondria, cell adhesion, cytoskeleton, and extracellular matrix components [34, 37]. Interestingly, our electrophysiological assay showed that the Ca^2+^ transient duration in *RRAGD* ^WT/p.Ser76Leu^ hiPSC‐CMs was significantly longer than those in *RRAGD* ^WT/WT^ hiPSC‐CMs, suggesting impaired Ca^2+^ handling in mutant cardiomyocytes. Of note, this Ca^2+^ transient measurement was performed on one batch of differentiation and warrants being repeated in the future. In previous DCM models, TAB2 deficiency in hiPSC‐CMs caused an increased time to peak (representing the time of calcium release into the cytosol) and prolonged Ca^2+^ transient duration (representing the time to release and remove calcium from the cytosol), while the p.(Arg173Trp) variant in the *TNNT2* gene in patient‐specific hiPSC‐CMs caused smaller Ca^2+^ amplitude without a change in timing [38, 39]. In both studies, reduced contractility was observed [38, 39]. We did not investigate if the cardiomyocyte contractility was compromised in our cells. Nevertheless, due to the changes in the expression of genes related to cytoskeleton organization, muscle development, and impaired Ca^2+^ handling, we hypothesize that the *RRAGD* p.(Ser76Leu) variant is causing the cardiomyocytes to undergo either remodeling or dedifferentiation to less muscle‐like cells.

To uncover the consequences of the *RRAGD* p.(Ser76Leu) variant on mTORC1 signaling, we used HeLa cell lines that stably overexpress *RRAGD* WT or p.(Ser76Leu) and demonstrated that both canonical and non‐canonical mTORC1 responses to amino acid signaling are impaired in the mutant cell line. These results indicate that the *RRAGD* p.(Ser76Leu) variant diminishes the mTORC1 response to amino acid starvation, leading to its activation [1, 2]. Furthermore, we showed that this entire process relies on mTOR, as inhibiting mTOR with Torin1 completely reduced the phosphorylation levels of S6K, 4E‐BP1, and TFEB and resulted in TFEB nuclear localization. Interestingly, we also observed higher total TFEB expression in *RRAGD* WT and p.(Ser76Leu) cells than in mock cells, as did Sambri et al. [2]. In our current study, we showed that this increase was not due to changes in TFEB mRNA levels and suggested an increased protein expression. Indeed, this could be due to increased TFEB interactions with 14‐3‐3 protein, which was previously observed when ADKH‐*RRAGD* variants are expressed in cells [2]. TFEB phosphorylation at the Ser‐211 site increases TFEB interaction with 14‐3‐3 protein, thus preventing its nuclear translocation [17].

Our findings in *RRAGD* ^WT/p.(Ser76Leu)^ hiPSC‐CMs showed normal mTORC1 signaling to amino acids stimulation and starvation, which included normal RRAGD‐LAMP1 co‐localization, and normal TFEB phosphorylation and subcellular localization. In line with these data, no genes involved in mTOR, lysosomal biogenesis, autophagy, or mitochondrial pathways were enriched in our *RRAGD* ^WT/p.Ser76Leu^ cardiomyocytes based on the bulk RNA‐seq results. Interestingly, the same *RRAGD* variant had previously been shown to impair TFEB's response to pharmacologically induced lysosomal damage in hiPSC‐CMs [2]. Moreover, *RRAGC* variants have been associated with DCM in patients; at the cellular level, TFEB dysfunctions were reported [24, 25]. The normal mTORC1 signaling response to amino acid removal in our mutant cardiomyocyte model is, however, as expected, given the immature nature of 2D‐cultured hiPSC‐CMs [40]. During development, the level of mTORC1 activation in cardiomyocytes changes, with its activity being high during fetal stages and low after birth [41, 42, 43]. This might be due to the role of mTORC1 signaling in supporting cell proliferation and regeneration, and postnatally, it is known that cardiomyocytes have limited capacity to proliferate and regenerate [21, 44, 45, 46]. Considering that 2D‐cultured hiPSC‐CMs are early embryonic‐like and our findings suggest a dedifferentiating phenotype in the mutant cardiomyocytes, we hypothesized that the *RRAGD* variants maintain cardiomyocytes in an immature state, and this causes cardiac dysfunction after birth in our ADKH‐*RRAGD* patients. Indeed, 80% of ADKH‐*RRAGD* patients with the p.(Ser76Leu) variant who developed DCM exhibited symptoms between the ages of 3 and 32, when cardiomyocytes are generally considered mature [1, 4]. Although it is not known if cardiac dysfunction already manifests during prenatal development, our results indicate that cardiomyocyte maturity is an important factor to consider for future research.

In ADKH‐*RRAGD*, mTOR inhibition has been proposed as a potential therapy [1, 26]. However, the specificity and efficacy of mTOR inhibition in managing this disease remain the primary concern in prescribing this drug to patients [47, 48]. Indeed, while we have previously demonstrated that rapamycin treatment improved cardiac function in zebrafish embryos overexpressing *RRAGD*, we could not confirm if mTORC1 overactivation was present [26]. Regardless, the critical function of mTOR in DCM progression has been demonstrated in mice as cardiomyocyte‐specific inducible deletion of *Mtor* or *Raptor* induced DCM and reduced mortality [21, 22, 49, 50]. Lower phosphorylation of 4E‐BP1 and S6K, increased autophagy, and increased apoptosis were thought to be causative of the compromised cardiac functions in these models [21, 22, 49, 50]. In mouse models with preexisting DCM, pharmacological inhibition of mTORC1 improved cardiac function [51, 52]. However, this could mean that mTOR inhibition can improve DCM symptoms even in cases where mTOR dysregulation is not observed, suggesting symptomatic relief rather than targeting the cause. Previously, rapamycin treatment has been shown to benefit a DCM patient with severe heart failure due to reduced ejection fraction carrying *LMNA* p.(Glu161Lys) variant [53]. In the patient's cardiac biopsies, a higher mTOR phosphorylation compared to a healthy control was observed [53]. Six months of rapamycin treatment improved ejection fraction and the New York Heart Association (NYHA) functional classification from class IV to class II in this patient [53]. In this case, using rapamycin as a last resort was justified within the framework of personalized medicine [53]. Therefore, for ADKH‐*RRAGD* patients, it is essential to first elucidate the molecular etiology of the disease to determine whether mTORC1 activation is the primary pathogenic driver before evaluating the potential benefits of mTOR inhibition.

This study has several strengths and limitations. We are the first to generate an ADKH‐*RRAGD* hiPSC‐CMs model. In these cardiomyocytes, bulk RNA‐seq and Ca^2+^ transient assay results suggested that the 2D‐cultured *RRAGD* ^WT/p.(Ser76Leu)^ hiPSC‐CMs were less differentiated than *RRAGD* ^WT/WT^ hiPSC‐CMs. In cardiomyocytes, dedifferentiation signifies a cellular event where cardiomyocytes revert to a less differentiated state accompanied by cytoskeletal and contractile apparatus reorganization, the re‐expression of fetal genes, and changes in energy metabolism [54, 55]. Therefore, future studies should aim to evaluate if *RRAGD* ^WT/p.(Ser76Leu)^ hiPSC‐CMs are dedifferentiated, for example, by measuring the proliferating population and confirming if the changes in muscle development and cytoskeleton gene expression are followed by functional effects such as altered muscle development and cytoskeletal reorganization in these cardiomyocytes. An important limitation of our experiments is the immaturity of the hiPSC‐derived cardiomyocyte model, a well‐known issue in the field [56, 57]. To address this, future investigations should focus on enhancing the maturity of *RRAGD* ^WT/p.Ser76Leu^ hiPSC‐CMs, for instance, by utilizing 3D cultures such as engineered heart tissue (EHT), cardiac microtissues, or organoids, which more accurately mimic adult in vivo cardiomyocytes (reviewed in [58]). Advancing the maturation of hiPSC‐CMs through 3D cultures will be a valuable next step for investigating the molecular mechanisms underlying ADKH‐*RRAGD* and how variants in this gene contribute to DCM development in patients. Alternatively, cultured human myocardial slices are a fully mature model that can be very useful, although studying the effects of variants requires either successful genetic modification of the slices, or the availability of patient tissue obtained during cardiac surgeries such as heart transplantation or LVAD implantation.

In conclusion, this study demonstrated that the *RRAGD* p.(Ser76Leu) variant, the most prevalent ADKH‐*RRAGD* variant identified to date, induces mTORC1 activity resulting in dysregulation of both non‐canonical and canonical targets. Bulk RNA‐seq and Ca^2+^ transient measurement indicate less differentiated cardiomyocytes due to this variant, hinting at early cardiac dysfunction in ADKH‐*RRAGD* patients. Future studies should examine how *RRAGD* variants affect mTORC1 signaling and cellular processes in animal models or patient biopsies to further confirm the molecular mechanisms underlying DCM in ADKH‐*RRAGD*. Understanding the molecular mechanisms of ADKH‐*RRAGD* would be of utmost value in providing treatment options for patients.

## Author Contributions

Planned experiments: Anastasia Adella, Sara B. van Katwijk, Pieter A. Leermakers, Teun P. de Boer, Eva van Rooij, and Jeroen H. F. de Baaij; performed experiments: Anastasia Adella, Sara B. van Katwijk, Pieter A. Leermakers, Willem B. van Ham, Hesther de Ruiter, Judita Ilgutytė, Suzanne Hendrickx, and Levi Nijland; analyzed data: Anastasia Adella, Sara B. van Katwijk, Pieter A. Leermakers, Willem B. van Ham, Hesther de Ruiter, Judita Ilgutytė, Suzanne Hendrickx, and Levi Nijland; contributed reagents or other essential material: Teun P. de Boer, Eva van Rooij, Joost G. J. Hoenderop, and Jeroen H. F. de Baaij; wrote the paper: Anastasia Adella, revised the paper: all authors, supervision: Pieter A. Leermakers, Teun P. de Boer, Eva van Rooij, Joost G. J. Hoenderop, and Jeroen H. F. de Baaij, funding: Teun P. de Boer, Eva van Rooij, Joost G. J. Hoenderop, and Jeroen H. F. de Baaij.

## Funding

This work was supported by EC | European Research Council (ERC) (101040682), Nederlandse Organisatie voor Wetenschappelijk Onderzoek (NWO), (OCENW.M.21.022, VIDI 391 09150172110040).

## Conflicts of Interest

The authors declare no conflicts of interest.

## Supporting information


**Table S1:** List of primers. ^$^DNA template for CRISPR‐Cas9. Mutation site (*RRAGD* c.227C>T) is underlined. *Primers were used to amplify amplicons through PCR, and the forward primers were also used for Sanger sequencing.
**Table S2:** Differentially expressed variants between *RRAGD* ^WT/p.(Ser76Leu)^ and *RRAGD* ^WT/WT^ hiPSCs following whole genome sequencing. List of unique genetic variants between *RRAGD* ^WT/p.(Ser76Leu)^ and *RRAGD* ^WT/WT^ hiPSCs after filtering for variants that were non‐synonymous, in an exon or splice acceptor/donor region, and have > 10 variation.reads. The sequencing produced a quality score of over 92% bases above Q30.
**Table S3:** Differentially expressed genes between *RRAGD* ^WT/p.(Ser76Leu)^ and *RRAGD* ^WT/WT^ hiPSC‐CMs following bulk RNA‐seq. Log2FC: Log2 fold change. *p*_adj_: adjusted *p*‐value.
**Table S4:** *RRAGD* ^WT/p.S76L^ hiPSC‐CMs differentially expressed genes vs. DCM. List of genes differentially expressed in our bulk RNA‐seq data that overlapped with genes specific to DCM patients in Sweet et al. Log2FC: Log2 fold change, *p*_adj_: adjusted *p*‐value.

**Figure S1:** mTOR inhibition by rapamycin suppressed S6K and 4E‐BP1 phosphorylation but not TFEB in *RRAGD* p.(Ser76Leu) T‐REx HeLa cells. (A‐C) T‐REx HeLa cells stably overexpressing GFP (mock), GFP‐*RRAGD* WT (WT), and GFP‐*RRAGD* p.(Ser76Leu) (S76L) were exposed to amino acids stimulation using amino acids‐containing medium (+AA, gray bars) or amino acids‐deprived medium (−AA, blue bars) and rapamycin or DMSO. (A) Representative immunoblots of S6K, p‐S6K, TFEB, p‐TFEB, 4E‐BP1, p‐4E‐BP1, GFP, and GAPDH following treatment. (B, C) Quantification of (B) phosphorylated TFEB/total TFEB ratio and (C) phosphorylated TFEB/GAPDH ratio (mean ± SEM from three independent experiments, normalized to the DMSO mock +AA condition). Two‐way ANOVA followed by Šídák's multiple comparison test where the effects of DMSO and rapamycin treatment were compared within each genotype, and each amino acids treatment group. The normality of the distribution was tested using a Q‐Q plot.

**Figure S2:** CRISPR‐Cas9‐mediated generation of *RRAGD* ^WT/WT^ and *RRAGD* ^WT/p.(Ser76Leu)^ hiPSCs. (A) T7 endonuclease assay in HEK293 cells gDNA transiently transfected with either one of the six gRNAs, only transfection agent (i.e., PEI), or cells without any treatment (non‐treated or NT). gRNA #5 was picked for subsequent steps. Red arrows indicate DNA fragments cut by T7 endonuclease. (B) Sanger sequencing results of the top three predicted off‐target sites on gRNA #5. (C, D) Representative immunofluorescence images of pluripotency markers SOX‐2, OCT3/4, and NANOG (green), and DAPI counterstain (blue) in (C) *RRAGD* ^W*T*/p.(Ser76Leu)^ and (D) *RRAGD* ^WT/WT^ hiPSCs clones. Scale bars indicate 20 μm. (E, F) Karyo‐sequencing profiles of (E) *RRAGD* ^WT/p.(Ser76Leu)^ and (F) *RRAGD* ^WT/WT^ hiPSCs clones.

**Figure S3:** Enriched gene ontology (GO) terms within the bulk RNA‐seq differentially expressed gene list. (A–C) Bubble plots of top enriched (A) biological process, (B) molecular function, and (C) cellular component GO terms in *RRAGD* ^WT/p.(Ser76Leu)^ hiPSC‐CMs. The terms have been filtered for those relevant in cardiomyocytes. The ranking of the terms was based on fold enrichment. The intensity of the bubble color is based on −log_10_ false discovery rate (FDR); red shows upregulated pathways, and blue shows downregulated pathways. The bubble size indicates the number of genes in each pathway (count).

**Figure S4:** Validation of bulk RNA‐seq. (A) mRNA expression of differentially expressed genes associated with cardiomyopathies in *RRAGD* ^WT/WT^ (WT; gray bars) and *RRAGD* ^WT/p.(Ser76Leu)^ (S76L; blue bars) hiPSC‐CMs. Mean ± SEM from three independent differentiations. Two‐way ANOVA followed by Šídák's multiple comparison test was performed. The normality of the distribution was tested using a Q‐Q plot. (B) Representative immunoblots of p‐Akt, Akt, p‐TAZ, TAZ, RagD, and vinculin in *RRAGD* ^WT/WT^ and *RRAGD* ^WT/p.(Ser76Leu)^ hiPSC‐CMs. (C‐D) Quantification of (C) Akt phosphorylation and (D) TAZ phosphorylation immunoblots in *RRAGD* ^WT/WT^ (WT; gray bars) and *RRAGD* ^WT/p.(Ser76Leu)^ (S76L; blue bars) hiPSC‐CMs. Mean ± SEM from three independent differentiations, normalized to the WT. One‐tailed unpaired *t*‐test was performed.

## Data Availability

All data presented in this manuscript and the Supporting Information for external review are available in the Radboud Data Repository https://data.ru.nl/login/reviewer‐12460941/OZNSMULIIOILSRVLYQRNO6L6LO74OQTWHNCBXMQ. The RNA‐seq data are available from the Gene Expression Omnibus (GEO) using the accession number GSE323990.
