# Supplementary material for: RRAGD p.(Ser76Leu) Variant Causes Dysregulated Expression of Muscle Development and Cytoskeleton Genes in Cardiomyocytes
Source: FASEB J. 2026 Jun 22;40(12):e72070. doi: 10.1096/fj.202501099RR (PMC13285898; doi:10.1096/fj.202501099RR)
Supplement: Supplementary file 1 — Table S1: List of primers. $DNA template for CRISPR‐Cas9. Mutation site (RRAGD c.227C>T) is underlined. *Primers were used to amplify amplicons through PCR, and the forward primers were also used for Sanger sequencing. Table S2: Differentially expressed variants between RRAGD WT/p.(Ser76Leu) and RRAGD WT/WT hiPSCs following whole genome sequencing. List of unique genetic variants between RRAGD WT/p.(Ser76Leu) and RRAGD WT/WT hiPSCs after filtering for variants that were non‐synonymous, in an exon or splice acceptor/donor region, and have > 10 variation.reads. The sequencing produced a quality score of over 92% bases above Q30. Table S3: Differentially expressed genes between RRAGD WT/p.(Ser76Leu) and RRAGD WT/WT hiPSC‐CMs following bulk RNA‐seq. Log2FC: Log2 fold change. p adj: adjusted p‐value. Table S4: RRAGD WT/p.S76L hiPSC‐CMs differentially expressed genes vs. DCM. List of genes differentially expressed in our bulk RNA‐seq data that overlapped with genes specific to DCM patients in Sweet et al. Log2FC: Log2 fold change, p adj: adjusted p‐value. [file FSB2-40-e72070-s002.docx]

**Supplemental materials**

***RRAGD* p.(Ser76Leu) variant causes dysregulated expression of
muscle development and cytoskeleton genes in cardiomyocytes**

Anastasia Adella^1#^, Sara B. van Katwijk^1#^, Pieter A. Leermakers^1^, Willem B. van Ham^2^, Hesther de Ruiter^3^, Judita Ilgutytė^1^, Suzanne Hendrickx^1^, Levi Nijland^1^, Teun P. de Boer^2^, Eva van Rooij^3, 4^, Joost G.J. Hoenderop^1^, Jeroen H.F. de Baaij^1*^

^1^ Department of Medical BioSciences, Radboudumc, Nijmegen, The Netherlands

^2^ Department of Medical Physiology, University Medical Center Utrecht, Utrecht, The Netherlands

^3^ Hubrecht Institute, Royal Netherlands Academy of Arts and Sciences (KNAW) and University Medical Center Utrecht, Utrecht, The Netherlands

^4^ Division Heart and Lungs, Department of Cardiology, University Medical Center Utrecht, Utrecht, The Netherlands

# These authors contributed equally

* Corresponding author:

Jeroen H.F. de Baaij

Department of Medical BioSciences,

Radboudumc

P.O. Box 9101, 6500HB, Nijmegen, The Netherlands

Phone: +31- 24 361 7347, Email: [jeroen.debaaij@radboudumc.nl](mailto:jeroen.debaaij@radboudumc.nl)

**Supplementary Figure 1. mTOR inhibition by rapamycin suppressed S6K and 4E-BP1 phosphorylation but not TFEB in *RRAGD* p.(Ser76Leu) T-REx HeLa cells**. (A-C) T-REx HeLa cells stably overexpressing GFP (mock), GFP-*RRAGD* WT (WT), and GFP-*RRAGD* p.(Ser76Leu) (S76L) were exposed to amino acids stimulation using amino acids-containing medium (+AA, grey bars) or amino acids-deprived medium (-AA, blue bars) and rapamycin or DMSO. (A) Representative immunoblots of S6K, p-S6K, TFEB, p-TFEB, 4E-BP1, p-4E-BP1, GFP, and GAPDH following treatment. (B, C) Quantification of (B) phosphorylated TFEB/total TFEB ratio and (C) phosphorylated TFEB/GAPDH ratio (mean ± SEM from three independent experiments, normalized to the DMSO mock +AA condition). Two-way ANOVA followed by Šídák’s multiple comparison test where the effects of DMSO and rapamycin treatment were compared within each genotype, and each amino acids treatment group. The normality of the distribution was tested using a Q-Q plot.

**Supplementary Figure 2. CRISPR-Cas9-mediated generation of *RRAGD*^WT/WT^ and *RRAGD*^WT/p.(Ser76Leu)^ hiPSCs.** (A) T7 endonuclease assay in HEK293 cells gDNA transiently transfected with either one of the six gRNAs, only transfection agent (*i.e.*, PEI), or cells without any treatment (non-treated or NT). gRNA #5 was picked for subsequent steps. Red arrows indicate DNA fragments cut by T7 endonuclease. (B) Sanger sequencing results of the top three predicted off-target sites on gRNA #5. (C-D) Representative immunofluorescence images of pluripotency markers SOX-2, OCT3/4, and NANOG (green), and DAPI counterstain (blue) in (C) *RRAGD*^W^*^T^*^/p.(Ser76Leu)^ and (D) *RRAGD*^WT/WT^ hiPSCs clones. Scale bars indicate 20 µm. (E-F) Karyo-sequencing profiles of (E) *RRAGD*^WT/p.(Ser76Leu)^ and (F) *RRAGD*^WT/WT^ hiPSCs clones.

**Supplementary Figure 3. Enriched gene ontology (GO) terms within the bulk RNA-seq differentially expressed gene list.** (A-C) Bubble plots of top enriched (A) biological process, (B) molecular function, and (C) cellular component GO terms in *RRAGD*^WT/p.(Ser76Leu)^ hiPSC-CMs. The terms have been filtered for those relevant in cardiomyocytes. The ranking of the terms was based on fold enrichment. The intensity of the bubble color is based on -log_10_ false discovery rate (FDR); red shows upregulated pathways, and blue shows downregulated pathways. The bubble size indicates the number of genes in each pathway (count).

**Supplementary Figure 4. Validation of bulk RNA-seq.** (A) mRNA expression of differentially expressed genes associated with cardiomyopathies in *RRAGD*^WT/WT^ (WT; grey bars) and *RRAGD*^WT/p.(Ser76Leu)^ (S76L; blue bars) hiPSC-CMs. Mean ± SEM from three independent differentiations. Two-way ANOVA followed by Šídák’s multiple comparison test was performed. The normality of the distribution was tested using a Q-Q plot. (B) Representative immunoblots of p-Akt, Akt, p-TAZ, TAZ, RagD, and vinculin in *RRAGD*^WT/WT^ and *RRAGD*^WT/p.(Ser76Leu)^ hiPSC-CMs. (C-D) Quantification of (C) Akt phosphorylation and (D) TAZ phosphorylation immunoblots in *RRAGD*^WT/WT^ (WT; grey bars) and *RRAGD*^WT/p.(Ser76Leu)^ (S76L; blue bars) hiPSC-CMs. Mean ± SEM from three independent differentiations, normalized to the WT. One-tailed unpaired T-test was performed.

**Supplementary Table 1. List of primers.** ^$^DNA template for CRISPR-Cas9. Mutation site (*RRAGD* c.227C>T) is underlined. *Primers were used to amplify amplicons through PCR, and the forward primers were also used for Sanger sequencing.

| Usage | Target | Direction | Sequence (5’-3’) |
| --- | --- | --- | --- |
| Subcloning to pCINeo | All h*RRAGD* variants | Forward | GCACCGGTTGCCACCATGTACCCATACGATGTTCC |
|  |  | Reverse | GTGAATTCCTACAGCAGCACTCTAGGGGTCC |
| Subcloning to pcDNA5 | All h*RRAGD* variants | Forward | GCCGGTACCAGCCAGGTGCTGG |
|  |  | Reverse | GCCCTCGAGCTACAGCAGCACTCTAGG |
| Mutagenesis | h*RRAGD* p.(Ser76Leu) | Forward | GAGGAGAAGCGGCAAGTTGTCTATTCAGAAAGTTG |
|  |  | Reverse | CAACTTTCTGAATAGACAACTTGCCGCTTCTCCTC |
| RT-qPCR | h*ACTA2* | Forward | GGCAAGTGATCACCATCGGA |
| RT-qPCR |  | Reverse | GTGGTTTCATGGATGCCAGC |
| RT-qPCR | h*ADCY5* | Forward | AATGGGGACTACGAGGTGGA |
| RT-qPCR |  | Reverse | GGCCTTCTCTTCTTTCCGCT |
| RT-qPCR | h*ALPK3* | Forward | GTCCAGCCTGCTTGTGTTTG |
| RT-qPCR |  | Reverse | GATCTTGCACCCCTGGATGG |
| RT-qPCR | h*GAPDH* | Forward | GGAGTCAACGGATTTGGTCGTA |
| RT-qPCR |  | Reverse | GGCAACAATATCCACTTTACCAGAGT |
| RT-qPCR | h*MYL2* | Forward | GAATTCTTCTCGGGAGGCAGTG |
| RT-qPCR |  | Reverse | CGAACATGGAGAACACGTTGG |
| RT-qPCR | h*MYL3* | Forward | CAAGACAGGAAGAGCTCAATAC |
| RT-qPCR |  | Reverse | CCTCATAGGTGCCTGTGTCC |
| RT-qPCR | h*NPPA* | Forward | CCGTGAGCTTCCTCCTTTTA |
| RT-qPCR |  | Reverse | CCAAATGGTCCAGCAAATTC |
| RT-qPCR | h*RPLP0* | Forward | ACTCTGCATTCTCGCTTCCT |
| RT-qPCR |  | Reverse | AGGACTCGTTTGTACCCGTT |
| RT-qPCR | h*TFEB* | Forward | GGTGCAGTCCTACCTGGAG |
| RT-qPCR |  | Reverse | GTGGGCAGCAAACTTGTTCC |
| Cloning gRNA #5 to PX458 | h*RRAGD* p.(Ser76Leu) | Forward | CACCGCTCATGGGCCTGAGGAGAAG |
|  |  | Reverse | AAACCTTCTCCTCAGGCCCATGAGC |
| DNA template WT^$^ | h*RRAGD* WT |  | ATTCTCATTTTGTGGTTTCTCCAGTTCTGGACTTCAGTGACCCCTTCAGCACTGAAGTGAAGCCGAGAATTCTG*CTCA*TGGGCCTGAGGAGATCCGGCAAGTCGTCTATTCAGAAAGTTGTCTTTCA |
| DNA template KI^$^ | h*RRAGD* p.(Ser76Leu) |  | ATTCTCATTTTGTGGTTTCTCCAGTTCTGGACTTCAGTGACCCCTTCAGCACTGAAGTGAAGCCGAGAATTCTGCTCATGGGCCTGAGGAGATCCGGCAAGTTGTCTATTCAGAAAGTTGTCTTTCA |
| Genotyping* | All h*RRAGD* variants | Forward | TGGGAAGTATAGACCAGCGG |
|  |  | Reverse | TTTGTAGATGTTGACTAGCGGT |
| Off-target #1* | RNU-53P-HINT1 intergenic | Forward | CCACACATTGGGACACTCTAG |
|  |  | Reverse | GGAAAGCTTGGATGTGGATAC |
| Off-target #2* | ACSBG1 intron | Forward | GAGGACACAGGACACATTGTGC |
|  |  | Reverse | CTACAGGGAGCACTCCCACTG |
| Off-target #3* | RP11-68L18.1 exon | Forward | GAACTACATGACATGTTCTCCG |
|  |  | Reverse | GCTCTGGAACTGGCTTATATTC |

**Supplementary Table 2. Differentially expressed variants between *RRAGD*^WT/p.(Ser76Leu)^ and *RRAGD*^WT/WT^ hiPSCs following whole genome sequencing.** List of unique genetic variants between *RRAGD*^WT/p.(Ser76Leu)^ and *RRAGD*^WT/WT^ hiPSCs after filtering for variants that were non-synonymous, in an exon or splice acceptor/donor region, and have >10 variation.reads. The sequencing produced a quality score of over 92% bases above Q30.

| Gene.name | Gene.component | Variant.type | reads | variation.reads | Hgvsc | Hgvsp |
| --- | --- | --- | --- | --- | --- | --- |
| PRAMEF19 | EXON_REGION | Substitution | 50 | 16 | ENST00000376101.4:c.1261A>G | ENSP00000365269.2:p.Asn421Asp |
| NBPF1 | EXON_REGION | Substitution | 71 | 21 | ENST00000430580.6:c.2176A>G | ENSP00000474456.1:p.Lys726Glu |
| NBPF26 | EXON_REGION | Substitution | 32 | 11 | ENST00000611287.5:c.320C>T | ENSP00000482859.2:p.Thr107Met |
| NBPF26 | EXON_REGION | Substitution | 63 | 15 | ENST00000611287.5:c.592A>G | ENSP00000482859.2:p.Lys198Glu |
| HRNR | EXON_REGION | Substitution | 43 | 16 | ENST00000368801.4:c.6160G>A | ENSP00000357791.3:p.Gly2054Ser |
| HRNR | EXON_REGION | Substitution | 93 | 37 | ENST00000368801.4:c.5996A>T | ENSP00000357791.3:p.Gln1999Leu |
| FCGR2C | SD_SITE_CANONICAL | Substitution | 41 | 14 | ENST00000466542.6:c.798+1A>G |  |
| IGFN1 | EXON_REGION | Substitution | 47 | 13 | ENST00000335211.9:c.6172A>G | ENSP00000334714.4:p.Ser2058Gly |
| IGFN1 | EXON_REGION | Substitution | 47 | 12 | ENST00000335211.9:c.6179T>G | ENSP00000334714.4:p.Val2060Gly |
| OR2T3 | EXON_REGION | Substitution | 54 | 13 | ENST00000359594.3:c.892C>T | ENSP00000352604.2:p.Arg298Cys |
| OR2T34 | EXON_REGION | Substitution | 41 | 12 | ENST00000328782.3:c.464T>C | ENSP00000330904.2:p.Val155Ala |
| IGKV1-39 | EXON_REGION | Substitution | 31 | 11 | ENST00000498574.1:c.52C>T | ENSP00000419058.1:p.Arg18Ter |
| MUC20 | EXON_REGION | Substitution | 95 | 28 | ENST00000436408.6:c.8G>C | ENSP00000396774.1:p.Cys3Ser |
| MUC20 | EXON_REGION | Substitution | 47 | 11 | ENST00000436408.6:c.1396G>A | ENSP00000396774.1:p.Glu466Lys |
| MUC20 | EXON_REGION | Substitution | 49 | 12 | ENST00000436408.6:c.1398A>C | ENSP00000396774.1:p.Glu466Asp |
| MUC20 | EXON_REGION | Substitution | 46 | 11 | ENST00000436408.6:c.1399G>A | ENSP00000396774.1:p.Ala467Thr |
| MUC4 | EXON_REGION | Deletion | 29 | 11 | ENST00000463781.8:c.11882_11974del | ENSP00000417498.3:p.Val3961_Ser3992delinsAla |
| MUC4 | EXON_REGION | Deletion | 25 | 13 | ENST00000463781.8:c.11854_11901del | ENSP00000417498.3:p.Pro3952_Thr3967del |
| MUC4 | EXON_REGION | Substitution | 17 | 12 | ENST00000463781.8:c.11415G>C | ENSP00000417498.3:p.Gln3805His |
| MUC4 | EXON_REGION | Substitution | 42 | 15 | ENST00000463781.8:c.10624C>T | ENSP00000417498.3:p.Leu3542Phe |
| MUC4 | EXON_REGION | Substitution | 34 | 12 | ENST00000463781.8:c.8936A>T | ENSP00000417498.3:p.Asp2979Val |
| MUC4 | EXON_REGION | Substitution | 50 | 12 | ENST00000463781.8:c.7381G>C | ENSP00000417498.3:p.Asp2461His |
| MUC4 | EXON_REGION | Substitution | 84 | 25 | ENST00000463781.8:c.6637A>G | ENSP00000417498.3:p.Ser2213Gly |
| MUC4 | EXON_REGION | Substitution | 83 | 39 | ENST00000463781.8:c.6458T>C | ENSP00000417498.3:p.Val2153Ala |
| MUC4 | EXON_REGION | Substitution | 52 | 12 | ENST00000463781.8:c.6191C>G | ENSP00000417498.3:p.Pro2064Arg |
| AP3S1 | EXON_REGION | Substitution | 45 | 14 | ENST00000316788.12:c.473C>T | ENSP00000325369.7:p.Pro158Leu |
| HLA-C | EXON_REGION | Substitution | 53 | 17 | ENST00000376228.10:c.583T>C | ENSP00000365402.5:p.Tyr195His |
| HLA-C | EXON_REGION | Substitution | 57 | 22 | ENST00000376228.10:c.527C>T | ENSP00000365402.5:p.Ala176Val |
| HLA-C | EXON_REGION | Substitution | 48 | 13 | ENST00000376228.10:c.463C>A | ENSP00000365402.5:p.Arg155Ser |
| TMEM196 | EXON_REGION | Substitution | 36 | 15 | ENST00000405764.7:c.514G>A | ENSP00000384234.3:p.Val172Met |
| ZP3 | EXON_REGION | Substitution | 63 | 22 | ENST00000336517.8:c.790T>C | ENSP00000337310.4:p.Ser264Pro |
| ZP3 | EXON_REGION | Substitution | 64 | 23 | ENST00000336517.8:c.881G>C | ENSP00000337310.4:p.Arg294Thr |
| MUC3A | EXON_REGION | Substitution | 47 | 29 | ENST00000379458.9:c.5659G>A | ENSP00000368771.5:p.Val1887Ile |
| MUC3A | EXON_REGION | Substitution | 40 | 22 | ENST00000379458.9:c.5684C>A | ENSP00000368771.5:p.Thr1895Lys |
| MUC3A | EXON_REGION | Substitution | 32 | 16 | ENST00000379458.9:c.5696T>C | ENSP00000368771.5:p.Ile1899Thr |
| MUC3A | EXON_REGION | Insertion | 32 | 13 | ENST00000379458.9:c.6142_6143insGCA | ENSP00000368771.5:p.Ile2047_Thr2048insSer |
| MUC3A | EXON_REGION | Substitution | 100 | 61 | ENST00000379458.9:c.6668G>C | ENSP00000368771.5:p.Ser2223Thr |
| MUC3A | EXON_REGION | Substitution | 133 | 99 | ENST00000379458.9:c.6731C>G | ENSP00000368771.5:p.Thr2244Ser |
| MUC3A | EXON_REGION | Substitution | 58 | 30 | ENST00000379458.9:c.6785C>T | ENSP00000368771.5:p.Thr2262Ile |
| MUC3A | EXON_REGION | Substitution | 80 | 24 | ENST00000379458.9:c.8504T>C | ENSP00000368771.5:p.Met2835Thr |
| MUC12 | EXON_REGION | Substitution | 52 | 17 | ENST00000379442.7:c.1477G>A | ENSP00000368755.3:p.Ala493Thr |
| MUC12 | EXON_REGION | Substitution | 30 | 12 | ENST00000379442.7:c.8330G>A | ENSP00000368755.3:p.Arg2777His |
| FAM90A24P | EXON_REGION | Substitution | 89 | 23 | ENST00000520792.5:c.1007C>A | ENSP00000514267.1:p.Ala336Asp |
| SPATA31A1 | EXON_REGION | Substitution | 60 | 19 | ENST00000377647.6:c.1998C>A | ENSP00000366875.5:p.Asp666Glu |
| SPATA31A1 | EXON_REGION | Substitution | 41 | 12 | ENST00000377647.6:c.2072G>A | ENSP00000366875.5:p.Arg691Gln |
| CNTNAP3B | EXON_REGION | Substitution | 37 | 14 | ENST00000341990.8:c.1222C>T | ENSP00000481131.1:p.Leu408Phe |
| NUTM2A | EXON_REGION | Substitution | 66 | 21 | ENST00000381689.4:c.478G>A | ENSP00000371107.3:p.Ala160Thr |
| POLL | EXON_REGION | Substitution | 45 | 22 | ENST00000299206.8:c.1205C>A | ENSP00000299206.4:p.Ala402Glu |
| DRD4 | EXON_REGION | Substitution | 42 | 16 | ENST00000176183.6:c.860A>C | ENSP00000176183.5:p.Gln287Pro |
| MUC6 | EXON_REGION | Substitution | 44 | 11 | ENST00000421673.7:c.6197T>C | ENSP00000406861.2:p.Val2066Ala |
| MUC6 | EXON_REGION | Substitution | 44 | 11 | ENST00000421673.7:c.6196G>C | ENSP00000406861.2:p.Val2066Leu |
| MUC6 | EXON_REGION | Substitution | 115 | 44 | ENST00000421673.7:c.6079A>C | ENSP00000406861.2:p.Thr2027Pro |
| MUC6 | EXON_REGION | Substitution | 115 | 45 | ENST00000421673.7:c.6077T>C | ENSP00000406861.2:p.Ile2026Thr |
| MUC6 | EXON_REGION | Substitution | 95 | 26 | ENST00000421673.7:c.5228C>G | ENSP00000406861.2:p.Ala1743Gly |
| MUC6 | EXON_REGION | Substitution | 106 | 32 | ENST00000421673.7:c.4868T>C | ENSP00000406861.2:p.Leu1623Ser |
| MUC6 | EXON_REGION | Substitution | 88 | 31 | ENST00000421673.7:c.4813C>A | ENSP00000406861.2:p.Pro1605Thr |
| MUC6 | EXON_REGION | Substitution | 87 | 24 | ENST00000421673.7:c.4789A>C | ENSP00000406861.2:p.Lys1597Gln |
| MUC6 | EXON_REGION | Substitution | 60 | 27 | ENST00000421673.7:c.4619C>A | ENSP00000406861.2:p.Thr1540Asn |
| MUC6 | EXON_REGION | Substitution | 62 | 27 | ENST00000421673.7:c.4615C>A | ENSP00000406861.2:p.Pro1539Thr |
| MUC6 | EXON_REGION | Substitution | 63 | 27 | ENST00000421673.7:c.4609G>A | ENSP00000406861.2:p.Val1537Ile |
| MUC6 | EXON_REGION | Insertion | 56 | 21 | ENST00000421673.7:c.4585_4586insTG | ENSP00000406861.2:p.Thr1529MetfsTer64 |
| MUC6 | EXON_REGION | Substitution | 54 | 20 | ENST00000421673.7:c.4583A>C | ENSP00000406861.2:p.His1528Pro |
| MUC6 | EXON_REGION | Deletion | 53 | 18 | ENST00000421673.7:c.4577_4578del | ENSP00000406861.2:p.His1526LeufsTer10 |
| MUC6 | EXON_REGION | Substitution | 51 | 16 | ENST00000421673.7:c.4564C>T | ENSP00000406861.2:p.Pro1522Ser |
| MUC6 | EXON_REGION | Substitution | 39 | 12 | ENST00000421673.7:c.4511C>T | ENSP00000406861.2:p.Pro1504Leu |
| MUC5AC | EXON_REGION | Substitution | 44 | 21 | ENST00000621226.2:c.6873A>C | ENSP00000485659.1:p.Arg2291Ser |
| MUC5AC | EXON_REGION | Substitution | 38 | 18 | ENST00000621226.2:c.6884C>T | ENSP00000485659.1:p.Ala2295Val |
| MUC5AC | EXON_REGION | Substitution | 40 | 18 | ENST00000621226.2:c.6886T>C | ENSP00000485659.1:p.Ser2296Pro |
| MUC5AC | EXON_REGION | Substitution | 41 | 18 | ENST00000621226.2:c.6889C>A | ENSP00000485659.1:p.Pro2297Thr |
| MUC5AC | EXON_REGION | Substitution | 41 | 18 | ENST00000621226.2:c.6892G>A | ENSP00000485659.1:p.Ala2298Thr |
| MUC5AC | EXON_REGION | Substitution | 45 | 16 | ENST00000621226.2:c.6902C>T | ENSP00000485659.1:p.Thr2301Ile |
| MUC5AC | EXON_REGION | Deletion | 51 | 15 | ENST00000621226.2:c.6913_6914del | ENSP00000485659.1:p.Gly2305LysfsTer18 |
| MUC5AC | EXON_REGION | Insertion | 52 | 15 | ENST00000621226.2:c.6917_6918insAC | ENSP00000485659.1:p.Asn2306LysfsTer20 |
| KRTAP5-1 | EXON_REGION | Substitution | 36 | 14 | ENST00000382171.2:c.523G>A | ENSP00000371606.2:p.Gly175Arg |
| OR5L2 | EXON_REGION | Substitution | 36 | 13 | ENST00000378397.1:c.18C>A | ENSP00000367650.1:p.Cys6Ter |
| OR8U1 | EXON_REGION | Substitution | 41 | 11 | ENST00000302270.1:c.316A>G | ENSP00000304188.1:p.Thr106Ala |
| OR8U1 | EXON_REGION | Insertion | 41 | 11 | ENST00000302270.1:c.325_326insCGGC | ENSP00000304188.1:p.Ile109ThrfsTer14 |
| OR8U1 | EXON_REGION | Deletion | 41 | 11 | ENST00000302270.1:c.327_330del | ENSP00000304188.1:p.Ile109MetfsTer26 |
| OR9G1 | EXON_REGION | Substitution | 65 | 14 | ENST00000642097.1:c.292T>G | ENSP00000493255.1:p.Cys98Gly |
| OR8G2P | EXON_REGION | Substitution | 54 | 12 | ENST00000412796.2:c.191G>A | ENSP00000502842.1:p.Ser64Asn |
| OR8G2P | EXON_REGION | Substitution | 52 | 12 | ENST00000412796.2:c.193G>A | ENSP00000502842.1:p.Gly65Ser |
| OR8G2P | EXON_REGION | Substitution | 53 | 13 | ENST00000412796.2:c.200C>T | ENSP00000502842.1:p.Ser67Phe |
| PRB1 | EXON_REGION | Substitution | 58 | 19 | ENST00000500254.6:c.263G>A | ENSP00000420826.2:p.Arg88Gln |
| NOC4L | SD_SITE_CANONICAL | Deletion | 23 | 21 | ENST00000330579.6:c.901+18_901+61del |  |
| IGHV3-48 | EXON_REGION | Substitution | 45 | 14 | ENST00000390624.3:c.220G>A | ENSP00000375033.2:p.Gly74Ser |
| IGHV4-59 | EXON_REGION | Substitution | 50 | 12 | ENST00000390629.3:c.206A>G | ENSP00000375038.2:p.Tyr69Cys |
| IGHV4-59 | EXON_REGION | Substitution | 49 | 12 | ENST00000390629.3:c.205T>C | ENSP00000375038.2:p.Tyr69His |
| IGHV4-59 | EXON_REGION | Substitution | 54 | 15 | ENST00000390629.3:c.178C>G | ENSP00000375038.2:p.Pro60Ala |
| GOLGA6L2 | EXON_REGION | Substitution | 12 | 11 | ENST00000567107.6:c.2165C>G | ENSP00000454407.1:p.Ala722Gly |
| GOLGA6L2 | EXON_REGION | Insertion | 20 | 16 | ENST00000567107.6:c.2158_2159insGATATGGGAG | ENSP00000454407.1:p.Ser720Ter |
| GOLGA6L2 | EXON_REGION | Substitution | 20 | 19 | ENST00000567107.6:c.2158T>A | ENSP00000454407.1:p.Ser720Thr |
| TPSD1 | EXON_REGION | Substitution | 57 | 14 | ENST00000211076.5:c.428A>G | ENSP00000211076.3:p.His143Arg |
| TPSD1 | EXON_REGION | Substitution | 56 | 14 | ENST00000211076.5:c.430A>G | ENSP00000211076.3:p.Ile144Val |
| CLEC18B | EXON_REGION | Substitution | 74 | 24 | ENST00000339953.9:c.517A>G | ENSP00000341051.5:p.Thr173Ala |
| LRRC37B | EXON_REGION | Substitution | 64 | 19 | ENST00000341671.12:c.2144C>T | ENSP00000340519.7:p.Ala715Val |
| USP32 | EXON_REGION | Substitution | 61 | 23 | ENST00000300896.9:c.2803C>T | ENSP00000300896.3:p.Arg935Trp |
| ZNF729 | EXON_REGION | Substitution | 35 | 16 | ENST00000601693.2:c.2088C>A | ENSP00000469582.1:p.Phe696Leu |
| DHX34 | EXON_REGION | Substitution | 31 | 13 | ENST00000328771.9:c.640C>G | ENSP00000331907.4:p.Arg214Gly |
| FAM90A27P | EXON_REGION | Substitution | 55 | 17 | ENST00000593323.2:c.1529T>C | ENSP00000513421.1:p.Met510Thr |
| LILRB3 | EXON_REGION | Substitution | 55 | 11 | ENST00000245620.13:c.181G>C | ENSP00000245620.9:p.Asp61His |
| LILRB3 | EXON_REGION | Substitution | 53 | 17 | ENST00000245620.13:c.176A>G | ENSP00000245620.9:p.Gln59Arg |
| IGLV5-48 | EXON_REGION | Substitution | 53 | 15 | ENST00000390293.1:c.115C>T | ENSP00000374828.2:p.Leu39Phe |
| IGLV5-48 | EXON_REGION | Substitution | 44 | 12 | ENST00000390293.1:c.152G>C | ENSP00000374828.2:p.Ser51Thr |
| IGLV5-45 | EXON_REGION | Substitution | 45 | 11 | ENST00000390296.2:c.61G>C | ENSP00000374831.2:p.Ala21Pro |
| TCN2 | EXON_REGION | Substitution | 34 | 14 | ENST00000698266.1:c.1292T>C | ENSP00000513637.1:p.Phe431Ser |
| RBMX | EXON_REGION | Substitution | 50 | 17 | ENST00000320676.11:c.1066G>T | ENSP00000359645.3:p.Gly356Trp |
| FBLIM1 | EXON_REGION | Substitution | 47 | 11 | ENST00000441801.6:c.1076G>T | ENSP00000416387.2:p.Gly359Val |
| FBLIM1 | EXON_REGION | Substitution | 52 | 13 | ENST00000441801.6:c.1089G>A | ENSP00000416387.2:p.Trp363Ter |
| NBPF1 | EXON_REGION | Substitution | 27 | 11 | ENST00000430580.6:c.1099A>G | ENSP00000474456.1:p.Lys367Glu |
| NBPF26 | EXON_REGION | Substitution | 37 | 19 | ENST00000620612.6:c.2803G>A | ENSP00000481542.4:p.Asp935Asn |
| NBPF20 | EXON_REGION | Substitution | 45 | 11 | ENST00000369373.9:c.93C>G | ENSP00000358380.6:p.Asn31Lys |
| IGFN1 | EXON_REGION | Substitution | 30 | 11 | ENST00000335211.9:c.6119C>T | ENSP00000334714.4:p.Ala2040Val |
| OR2T34 | EXON_REGION | Substitution | 33 | 11 | ENST00000328782.3:c.716G>A | ENSP00000330904.2:p.Arg239His |
| MUC20 | EXON_REGION | Substitution | 67 | 25 | ENST00000436408.6:c.1769C>T | ENSP00000396774.1:p.Pro590Leu |
| MUC20 | EXON_REGION | Substitution | 62 | 23 | ENST00000436408.6:c.1783G>A | ENSP00000396774.1:p.Ala595Thr |
| MUC4 | EXON_REGION | Substitution | 13 | 11 | ENST00000463781.8:c.11902T>C | ENSP00000417498.3:p.Ser3968Pro |
| MUC4 | EXON_REGION | Substitution | 52 | 17 | ENST00000463781.8:c.8800C>T | ENSP00000417498.3:p.Leu2934Phe |
| MUC4 | EXON_REGION | Substitution | 54 | 12 | ENST00000463781.8:c.8734C>A | ENSP00000417498.3:p.Pro2912Thr |
| MUC4 | EXON_REGION | Deletion | 38 | 36 | ENST00000463781.8:c.7005del | ENSP00000417498.3:p.Pro2336LeufsTer668 |
| FGF5 | EXON_REGION | Substitution | 41 | 23 | ENST00000312465.12:c.179C>T | ENSP00000311697.7:p.Ser60Phe |
| ERAP2 | SD_SITE_CANONICAL | Insertion | 20 | 15 | ENST00000379904.8:c.1368+3_1368+102dup |  |
| RRAGD | EXON_REGION | Substitution | 49 | 25 | ENST00000369415.9:c.227C>T | ENSP00000358423.4:p.Ser76Leu |
| USP42 | EXON_REGION | Substitution | 45 | 27 | ENST00000306177.10:c.3345C>A | ENSP00000301962.5:p.Ser1115Arg |
| TRGC1 | EXON_REGION | Substitution | 34 | 11 | ENST00000443402.6:c.484A>G | ENSP00000404817.2:p.Arg162Gly |
| PKD1L1 | EXON_REGION | Substitution | 34 | 17 | ENST00000289672.7:c.2067A>G | ENSP00000289672.2:p.Ile689Met |
| MUC3A | EXON_REGION | Substitution | 46 | 11 | ENST00000379458.9:c.4541A>T | ENSP00000368771.5:p.Asn1514Ile |
| MUC3A | EXON_REGION | Insertion | 46 | 11 | ENST00000379458.9:c.6686_6687insCGACTCGATCGTCACCAC | ENSP00000368771.5:p.Thr2229_Glu2230insAspSerIleValThrThr |
| MUC12 | EXON_REGION | Substitution | 31 | 13 | ENST00000379442.7:c.3659G>A | ENSP00000368755.3:p.Arg1220His |
| PABPC1 | EXON_REGION | Substitution | 59 | 17 | ENST00000318607.10:c.1549G>C | ENSP00000313007.5:p.Val517Leu |
| PABPC1 | EXON_REGION | Substitution | 54 | 18 | ENST00000318607.10:c.1516C>T | ENSP00000313007.5:p.Arg506Cys |
| PABPC1 | EXON_REGION | Substitution | 54 | 18 | ENST00000318607.10:c.1513G>A | ENSP00000313007.5:p.Val505Ile |
| PABPC1 | EXON_REGION | Substitution | 53 | 18 | ENST00000318607.10:c.1477C>T | ENSP00000313007.5:p.Arg493Cys |
| PABPC1 | SA_SITE_CANONICAL | Deletion | 69 | 17 | ENST00000318607.10:c.1447+1_1448-1del |  |
| PABPC1 | EXON_REGION | Substitution | 44 | 11 | ENST00000318607.10:c.1205C>T | ENSP00000313007.5:p.Pro402Leu |
| PABPC1 | EXON_REGION | Substitution | 51 | 12 | ENST00000318607.10:c.1120C>T | ENSP00000313007.5:p.Arg374Cys |
| PABPC1 | EXON_REGION | Substitution | 54 | 12 | ENST00000318607.10:c.1115A>G | ENSP00000313007.5:p.Glu372Gly |
| PABPC1 | EXON_REGION | Substitution | 56 | 12 | ENST00000318607.10:c.1093G>T | ENSP00000313007.5:p.Val365Leu |
| EPPK1 | EXON_REGION | Substitution | 47 | 16 | ENST00000568225.2:c.14107G>A | ENSP00000456124.2:p.Ala4703Thr |
| GPRIN2 | EXON_REGION | Substitution | 93 | 24 | ENST00000374314.6:c.1337G>A | ENSP00000363433.4:p.Arg446His |
| GPRIN2 | EXON_REGION | Substitution | 91 | 24 | ENST00000374314.6:c.1042G>T | ENSP00000363433.4:p.Val348Leu |
| AGAP9 | EXON_REGION | Substitution | 32 | 11 | ENST00000452145.6:c.1550G>A | ENSP00000392206.2:p.Arg517Gln |
| AGAP9 | EXON_REGION | Substitution | 27 | 13 | ENST00000452145.6:c.659C>T | ENSP00000392206.2:p.Pro220Leu |
| FRMPD2 | EXON_REGION | Substitution | 39 | 15 | ENST00000305531.3:c.2827A>G | ENSP00000307079.3:p.Ile943Val |
| NUTM2D | EXON_REGION | Substitution | 80 | 21 | ENST00000381697.7:c.103C>T | ENSP00000371116.1:p.His35Tyr |
| MUC6 | EXON_REGION | Substitution | 118 | 35 | ENST00000421673.7:c.5380C>A | ENSP00000406861.2:p.Pro1794Thr |
| MUC6 | EXON_REGION | Substitution | 121 | 38 | ENST00000421673.7:c.5272C>T | ENSP00000406861.2:p.His1758Tyr |
| MUC6 | EXON_REGION | Substitution | 121 | 42 | ENST00000421673.7:c.4918G>T | ENSP00000406861.2:p.Ala1640Ser |
| MUC5B | EXON_REGION | Substitution | 31 | 11 | ENST00000529681.5:c.6521A>G | ENSP00000436812.1:p.Asn2174Ser |
| KRTAP5-1 | EXON_REGION | Insertion | 28 | 14 | ENST00000382171.2:c.522_523insAGGGGCTGTGGCTCCTGTGGAGGCTGCAAGGGGGGCTGCGGTTCTTGTGGGGGCTCCAAG | ENSP00000371606.2:p.Lys174_Gly175insArgGlyCysGlySerCysGlyGlyCysLysGlyGlyCysGlySerCysGlyGlySerLys |
| DGKZ | EXON_REGION | Substitution | 50 | 11 | ENST00000318201.12:c.29C>A | ENSP00000320340.8:p.Ala10Asp |
| UNC93B1 | EXON_REGION | Substitution | 57 | 16 | ENST00000227471.7:c.1495G>A | ENSP00000227471.3:p.Val499Met |
| FAM186A | EXON_REGION | Substitution | 37 | 11 | ENST00000327337.6:c.4804C>T | ENSP00000329995.5:p.Pro1602Ser |
| PSPC1 | EXON_REGION | Substitution | 64 | 22 | ENST00000338910.9:c.1474A>G | ENSP00000343966.4:p.Met492Val |
| SKA3 | EXON_REGION | Substitution | 71 | 20 | ENST00000400018.7:c.1157A>G | ENSP00000382896.3:p.Lys386Arg |
| SKA3 | EXON_REGION | Substitution | 72 | 21 | ENST00000400018.7:c.1142C>T | ENSP00000382896.3:p.Thr381Ile |
| PABPC3 | EXON_REGION | Substitution | 51 | 13 | ENST00000281589.5:c.431A>G | ENSP00000281589.3:p.His144Arg |
| PABPC3 | EXON_REGION | Substitution | 53 | 15 | ENST00000281589.5:c.440C>T | ENSP00000281589.3:p.Thr147Ile |
| PABPC3 | EXON_REGION | Substitution | 52 | 15 | ENST00000281589.5:c.444C>G | ENSP00000281589.3:p.His148Gln |
| AHNAK2 | EXON_REGION | Substitution | 33 | 12 | ENST00000333244.6:c.3622A>G | ENSP00000353114.4:p.Thr1208Ala |
| IGHV3-66 | EXON_REGION | Substitution | 51 | 12 | ENST00000390632.2:c.91A>G | ENSP00000375041.2:p.Ile31Val |
| GOLGA6L10 | EXON_REGION | Deletion | 31 | 18 | ENST00000610657.2:c.835_855del | ENSP00000479362.1:p.Arg279_Leu285del |
| TPSAB1 | EXON_REGION | Substitution | 47 | 13 | ENST00000338844.8:c.68G>T | ENSP00000343577.3:p.Gly23Val |
| CLEC18C | EXON_REGION | Substitution | 36 | 12 | ENST00000314151.12:c.299T>C | ENSP00000326538.8:p.Leu100Pro |
| CCDC40 | EXON_REGION | Substitution | 62 | 21 | ENST00000374877.7:c.2891A>G | ENSP00000364011.3:p.His964Arg |
| EIF4A3 | SD_SITE_CANONICAL | Deletion | 31 | 11 | ENST00000647795.1:c.-68_-62+13del |  |
| PLIN4 | EXON_REGION | Substitution | 53 | 12 | ENST00000301286.5:c.2024T>C | ENSP00000301286.4:p.Val675Ala |
| KDM4B | EXON_REGION | Insertion | 29 | 21 | ENST00000159111.9:c.2901+62_2901+63insCGAGAGCATCACGGTGAGCTGTGGGGTGGGGCAGGGGGCGGGGGGAGGCTGGGAGCACAGCGACAACCTGTACCC |  |
| FCGBP | EXON_REGION | Substitution | 36 | 11 | ENST00000616721.6:c.7621G>C | ENSP00000481056.3:p.Val2541Leu |
| FCGBP | EXON_REGION | Substitution | 32 | 11 | ENST00000616721.6:c.5323A>G | ENSP00000481056.3:p.Thr1775Ala |
| FCGBP | EXON_REGION | Substitution | 38 | 11 | ENST00000616721.6:c.4601A>C | ENSP00000481056.3:p.Gln1534Pro |
| LILRB3 | EXON_REGION | Substitution | 63 | 16 | ENST00000245620.13:c.523G>C | ENSP00000245620.9:p.Gly175Arg |
| LILRB3 | EXON_REGION | Substitution | 72 | 21 | ENST00000245620.13:c.343C>A | ENSP00000245620.9:p.Leu115Met |
| LILRA6 | EXON_REGION | Substitution | 98 | 30 | ENST00000245621.6:c.1226T>A | ENSP00000245621.4:p.Phe409Tyr |
| LILRA6 | EXON_REGION | Substitution | 98 | 30 | ENST00000245621.6:c.1225T>C | ENSP00000245621.4:p.Phe409Leu |
| ICOSLG | EXON_REGION | Substitution | 47 | 11 | ENST00000400379.8:c.1325T>C | ENSP00000383230.3:p.Leu442Pro |
| KRTAP10-4 | EXON_REGION | Substitution | 55 | 12 | ENST00000400374.4:c.184C>T | ENSP00000383225.3:p.Arg62Cys |
| ARMCX4 | EXON_REGION | Substitution | 24 | 12 | ENST00000423738.5:c.5495G>A | ENSP00000404304.3:p.Gly1832Glu |
| WASH6P | EXON_REGION | Substitution | 76 | 19 | ENST00000359512.8:c.1201C>G | ENSP00000504557.1:p.Leu401Val |

**Supplementary Table 3. Differentially expressed genes between *RRAGD*^WT/p.(Ser76Leu)^ and *RRAGD*^WT/WT^ hiPSC-CMs following bulk RNA-seq.** Log2FC: Log2 fold change. Padj: adjusted p-value.

| Gene ID | hgnc_symbol | Mean count WT | Mean count S76L | Log2FC | p-value | padj |
| --- | --- | --- | --- | --- | --- | --- |
| ENSG00000136383 | ALPK3 | 2056.75 | 500 | -2.643519469 | 4.45E-97 | 7.02E-93 |
| ENSG00000112936 | C7 | 726.5 | 3799.75 | 1.768109681 | 1.04E-43 | 8.23E-40 |
| ENSG00000135842 | NIBAN1 | 1397.5 | 897.25 | -1.287690259 | 2.04E-17 | 1.07E-13 |
| ENSG00000153993 | SEMA3D | 22.25 | 148.25 | 2.055427677 | 4.01E-13 | 1.58E-09 |
| ENSG00000251129 | WWC2-AS1 | 46 | 236.25 | 1.612113228 | 6.51E-13 | 2.05E-09 |
| ENSG00000145741 | BTF3 | 494 | 1435.75 | 0.91179882 | 1.44E-12 | 3.78E-09 |
| ENSG00000152583 | SPARCL1 | 9.25 | 132.25 | 3.213681219 | 4.61E-12 | 1.04E-08 |
| ENSG00000186073 | CDIN1 | 516 | 400.75 | -0.927229837 | 1.87E-11 | 3.68E-08 |
| ENSG00000167785 | ZNF558 | 0 | 50 | 8.536510024 | 3.54E-11 | 6.21E-08 |
| ENSG00000134716 | CYP2J2 | 756.75 | 590.75 | -0.920794414 | 1.23E-10 | 1.94E-07 |
| ENSG00000197565 | COL4A6 | 814.5 | 2071.5 | 0.711896709 | 2.75E-10 | 3.95E-07 |
| ENSG00000005108 | THSD7A | 36.5 | 172.25 | 1.513620955 | 8.76E-10 | 0.00000115 |
| ENSG00000266472 | MRPS21 | 119.25 | 391.25 | 1.058511994 | 1.25E-09 | 0.00000152 |
| ENSG00000125730 | C3 | 3.25 | 152.5 | 0.02475256 | 2.54E-09 | 0.00000287 |
| ENSG00000272674 | PCDHB16 | 33.25 | 138.25 | 1.339431262 | 3.09E-09 | 0.00000325 |
| ENSG00000039068 | CDH1 | 3.25 | 83.25 | 0.037477287 | 5.02E-09 | 0.00000496 |
| ENSG00000278318 | ZNF229 | 0 | 29.25 | 7.565604678 | 5.44E-09 | 0.00000505 |
| ENSG00000108439 | PNPO | 75 | 41.5 | -1.371997047 | 1.35E-08 | 0.0000118 |
| ENSG00000120729 | MYOT | 5.25 | 53.25 | 2.55456972 | 1.93E-08 | 0.0000161 |
| ENSG00000173406 | DAB1 | 342.5 | 866.25 | 0.698018873 | 5.99E-08 | 0.000045 |
| ENSG00000197614 | MFAP5 | 8 | 113 | 0.040310508 | 5.85E-08 | 0.000045 |
| ENSG00000189221 | MAOA | 11.5 | 94.75 | 2.174514026 | 8.05E-08 | 0.0000577 |
| ENSG00000101638 | ST8SIA5 | 43 | 17 | -1.844412851 | 0.00000012 | 0.000082 |
| ENSG00000156466 | GDF6 | 160.5 | 490 | 0.922341612 | 0.000000134 | 0.0000881 |
| ENSG00000052850 | ALX4 | 8.5 | 62.5 | 2.099268331 | 0.000000178 | 0.00011245 |
| ENSG00000153707 | PTPRD | 1167.25 | 2682.75 | 0.536064543 | 0.000000201 | 0.00012219 |
| ENSG00000105894 | PTN | 706.75 | 1772.25 | 0.694591229 | 0.000000214 | 0.0001251 |
| ENSG00000070193 | FGF10 | 135.75 | 390.5 | 0.878072087 | 0.000000235 | 0.0001259 |
| ENSG00000077009 | NMRK2 | 59.75 | 23.25 | -1.859605883 | 0.000000239 | 0.0001259 |
| ENSG00000091136 | LAMB1 | 1335.5 | 3626.5 | 0.710194041 | 0.000000235 | 0.0001259 |
| ENSG00000175206 | NPPA | 3996 | 3282.5 | -0.82921057 | 0.000000316 | 0.00016114 |
| ENSG00000160808 | MYL3 | 9677.5 | 7296.25 | -0.913395877 | 0.000000473 | 0.00023326 |
| ENSG00000171617 | ENC1 | 40.5 | 180.25 | 1.34376473 | 0.000000793 | 0.00037931 |
| ENSG00000137261 | KIAA0319 | 44 | 151.25 | 1.080629249 | 0.000000846 | 0.00039276 |
| ENSG00000156253 | RWDD2B | 9.5 | 0 | -7.002290045 | 0.00000123 | 0.00055596 |
| ENSG00000113248 | PCDHB15 | 53.5 | 174.75 | 0.967307135 | 0.00000204 | 0.00089297 |
| ENSG00000125266 | EFNB2 | 168 | 556.75 | 0.967528846 | 0.00000322 | 0.00137355 |
| ENSG00000100461 | RBM23 | 273.75 | 249.25 | -0.66031649 | 0.0000035 | 0.00145271 |
| ENSG00000075340 | ADD2 | 355.5 | 278 | -0.820680109 | 0.00000439 | 0.00176707 |
| ENSG00000197893 | NRAP | 514 | 186.75 | -2.053327933 | 0.00000448 | 0.00176707 |
| ENSG00000079691 | CARMIL1 | 81.5 | 232.75 | 0.837448415 | 0.00000486 | 0.00178361 |
| ENSG00000118257 | NRP2 | 222 | 795 | 1.029828025 | 0.00000477 | 0.00178361 |
| ENSG00000206579 | XKR4 | 6.5 | 48 | 1.994779843 | 0.00000466 | 0.00178361 |
| ENSG00000108950 | FAM20A | 141.75 | 388.25 | 0.714175641 | 0.00000533 | 0.00186866 |
| ENSG00000213949 | ITGA1 | 102.25 | 315.75 | 0.866639512 | 0.00000528 | 0.00186866 |
| ENSG00000163430 | FSTL1 | 3320.5 | 8044.25 | 0.538837039 | 0.00000562 | 0.00192687 |
| ENSG00000184347 | SLIT3 | 742.25 | 1702 | 0.523442118 | 0.00000658 | 0.00220959 |
| ENSG00000091972 | CD200 | 24 | 132.5 | 1.708332817 | 0.00000748 | 0.00240674 |
| ENSG00000112414 | ADGRG6 | 95.5 | 299.75 | 0.961147358 | 0.00000778 | 0.00240674 |
| ENSG00000164764 | SBSPON | 1.5 | 47.25 | 3.738610914 | 0.00000768 | 0.00240674 |
| ENSG00000185847 | LINC01405 | 347.75 | 293.5 | -0.779167998 | 0.00000773 | 0.00240674 |
| ENSG00000182732 | RGS6 | 102 | 291 | 0.815191095 | 0.00000987 | 0.00299523 |
| ENSG00000145808 | ADAMTS19 | 94.5 | 315.25 | 1.057161754 | 0.0000143 | 0.00426327 |
| ENSG00000024422 | EHD2 | 47 | 240.5 | 1.451674842 | 0.0000152 | 0.00442837 |
| ENSG00000111245 | MYL2 | 13368 | 7567.75 | -1.397615899 | 0.0000159 | 0.00457028 |
| ENSG00000264230 | RNU4ATAC | 0 | 17.25 | 0.013456511 | 0.0000163 | 0.00458404 |
| ENSG00000092969 | TGFB2 | 179 | 458.75 | 0.659455094 | 0.0000174 | 0.00479514 |
| ENSG00000146530 | VWDE | 97.25 | 260 | 0.717416965 | 0.0000179 | 0.00479514 |
| ENSG00000288941 |  | 280.75 | 654.75 | 0.547647829 | 0.0000177 | 0.00479514 |
| ENSG00000187210 | GCNT1 | 9 | 48.5 | 1.561675717 | 0.0000197 | 0.00517896 |
| ENSG00000132470 | ITGB4 | 1 | 38 | 4.031167734 | 0.0000252 | 0.00635819 |
| ENSG00000134853 | PDGFRA | 101 | 397.25 | 1.059677657 | 0.000025 | 0.00635819 |
| ENSG00000197442 | MAP3K5 | 129.5 | 118 | -0.669065101 | 0.0000254 | 0.00635819 |
| ENSG00000067798 | NAV3 | 267.5 | 669.25 | 0.682043487 | 0.0000298 | 0.0073531 |
| ENSG00000137936 | BCAR3 | 68 | 221.25 | 1.00976701 | 0.0000355 | 0.00861396 |
| ENSG00000107796 | ACTA2 | 5196.5 | 14601.75 | 0.823175334 | 0.0000385 | 0.00909285 |
| ENSG00000169908 | TM4SF1 | 1.5 | 32 | 3.184117602 | 0.0000386 | 0.00909285 |
| ENSG00000099337 | KCNK6 | 394.75 | 963.5 | 0.594298132 | 0.0000423 | 0.00982376 |
| ENSG00000145934 | TENM2 | 200.25 | 620.75 | 0.930421415 | 0.000044 | 0.01006199 |
| ENSG00000153820 | SPHKAP | 1687.75 | 3747 | 0.461337379 | 0.000045 | 0.01014548 |
| ENSG00000173068 | BNC2 | 56 | 199.5 | 1.048056067 | 0.0000467 | 0.01039191 |
| ENSG00000270112 | CELF2-DT | 28.75 | 13 | -1.53411202 | 0.0000551 | 0.01207769 |
| ENSG00000125378 | BMP4 | 20.5 | 102 | 1.436529449 | 0.0000588 | 0.01271258 |
| ENSG00000147872 | PLIN2 | 78.5 | 53.25 | -0.980635223 | 0.0000599 | 0.01278257 |
| ENSG00000171357 | LURAP1 | 55 | 37 | -1.03736431 | 0.0000623 | 0.01311028 |
| ENSG00000275342 | FGD5P1 | 13 | 101 | 0.030981633 | 0.0000635 | 0.01318154 |
| ENSG00000181744 | DIPK2A | 477.75 | 1099.5 | 0.470782684 | 0.0000683 | 0.01400128 |
| ENSG00000109472 | CPE | 87 | 257.5 | 0.763555619 | 0.0000726 | 0.01468722 |
| ENSG00000143382 | ADAMTSL4 | 246.25 | 234.5 | -0.641285613 | 0.0000751 | 0.0150015 |
| ENSG00000151892 | GFRA1 | 139.5 | 381.75 | 0.766987407 | 0.0000786 | 0.01514495 |
| ENSG00000176658 | MYO1D | 96.25 | 271.75 | 0.777440437 | 0.0000771 | 0.01514495 |
| ENSG00000178031 | ADAMTSL1 | 132.25 | 392 | 0.789229725 | 0.0000787 | 0.01514495 |
| ENSG00000127824 | TUBA4A | 204 | 170.75 | -0.721186064 | 0.0000797 | 0.0151505 |
| ENSG00000138193 | PLCE1 | 879.5 | 1819.5 | 0.360986041 | 0.0000826 | 0.01552484 |
| ENSG00000186314 | PRELID2 | 253.25 | 574 | 0.513774196 | 0.0000966 | 0.0179461 |
| ENSG00000112379 | ARFGEF3 | 26.75 | 102.75 | 0.061517791 | 0.000100053 | 0.01836205 |
| ENSG00000171476 | HOPX | 329.5 | 262 | -0.896944836 | 0.000108619 | 0.01970495 |
| ENSG00000150782 | IL18 | 1.75 | 34.25 | 0.016769967 | 0.000118228 | 0.02120454 |
| ENSG00000163347 | CLDN1 | 7.25 | 47.5 | 1.731030719 | 0.000131309 | 0.02328602 |
| ENSG00000137571 | SLCO5A1 | 87.75 | 265.5 | 0.900635082 | 0.000137016 | 0.02376408 |
| ENSG00000150630 | VEGFC | 57.75 | 156.25 | 0.68261445 | 0.000136908 | 0.02376408 |
| ENSG00000131016 | AKAP12 | 684.25 | 1912.5 | 0.732734873 | 0.00014048 | 0.02409989 |
| ENSG00000111816 | FRK | 38.25 | 17.75 | -1.566147006 | 0.000142154 | 0.02412494 |
| ENSG00000101871 | MID1 | 278.25 | 632 | 0.456905427 | 0.000144528 | 0.02426682 |
| ENSG00000276476 | LINC00540 | 10.75 | 1.5 | -2.958512337 | 0.000146546 | 0.02434663 |
| ENSG00000105926 | PALS2 | 132.5 | 351.25 | 0.663754862 | 0.000149914 | 0.02443134 |
| ENSG00000162630 | B3GALT2 | 290 | 692.75 | 0.597237949 | 0.000150151 | 0.02443134 |
| ENSG00000055813 | CCDC85A | 38.75 | 115.25 | 0.779462381 | 0.000161567 | 0.02602046 |
| ENSG00000173175 | ADCY5 | 500.25 | 494 | -0.606844376 | 0.000168917 | 0.0269294 |
| ENSG00000088992 | TESC | 103.75 | 283.75 | 0.730063748 | 0.000175301 | 0.02695263 |
| ENSG00000111110 | PPM1H | 15.5 | 87.25 | 0.035016604 | 0.000171766 | 0.02695263 |
| ENSG00000140575 | IQGAP1 | 105.5 | 403.25 | 0.074202542 | 0.000176457 | 0.02695263 |
| ENSG00000165323 | FAT3 | 1016.5 | 2370.5 | 0.487761079 | 0.000177287 | 0.02695263 |
| ENSG00000289309 |  | 5.5 | 35.75 | 1.817452461 | 0.000177601 | 0.02695263 |
| ENSG00000116396 | KCNC4 | 280 | 274.75 | -0.573567763 | 0.000183283 | 0.0275501 |
| ENSG00000108797 | CNTNAP1 | 188.75 | 437 | 0.485654881 | 0.000193971 | 0.0288816 |
| ENSG00000243566 | RN7SL838P | 0 | 12.75 | 0.009931416 | 0.000200826 | 0.02962273 |
| ENSG00000136943 | CTSV | 72 | 256 | 0.915463818 | 0.000216084 | 0.03157834 |
| ENSG00000144278 | GALNT13 | 12.75 | 66.5 | 1.39670771 | 0.000230862 | 0.03342833 |
| ENSG00000108691 | CCL2 | 0.75 | 20.25 | 0.016806722 | 0.000258345 | 0.03706785 |
| ENSG00000154330 | PGM5 | 727.5 | 830.75 | -0.345460931 | 0.000277396 | 0.0392433 |
| ENSG00000205038 | PKHD1L1 | 0.75 | 72.5 | 0.006449277 | 0.000280966 | 0.0392433 |
| ENSG00000272079 | AC004233.3 | 44 | 31.75 | -0.944015201 | 0.0002792 | 0.0392433 |
| ENSG00000068976 | PYGM | 451.75 | 406.5 | -0.592041896 | 0.000284168 | 0.03934234 |
| ENSG00000011201 | ANOS1 | 167.5 | 392.25 | 0.481138439 | 0.000304804 | 0.04136784 |
| ENSG00000171812 | COL8A2 | 1.5 | 20.25 | 0.020768549 | 0.000306661 | 0.04136784 |
| ENSG00000175093 | SPSB4 | 174.25 | 163.5 | -0.652000477 | 0.000303225 | 0.04136784 |
| ENSG00000132199 | ENOSF1 | 245.75 | 229 | -0.604052852 | 0.00032828 | 0.04390891 |
| ENSG00000167363 | FN3K | 114.25 | 97.5 | -0.695779904 | 0.000335468 | 0.04449327 |
| ENSG00000143324 | XPR1 | 1325.5 | 2871.5 | 0.420704421 | 0.000345806 | 0.04502721 |
| ENSG00000180957 | PITPNB | 277.25 | 597.25 | 0.420053028 | 0.000348053 | 0.04502721 |
| ENSG00000182197 | EXT1 | 537.25 | 1268 | 0.506944262 | 0.000342806 | 0.04502721 |
| ENSG00000013016 | EHD3 | 745.75 | 1661.25 | 0.394671886 | 0.000357693 | 0.04523757 |
| ENSG00000156140 | ADAMTS3 | 64.25 | 203.25 | 0.858117354 | 0.000358278 | 0.04523757 |
| ENSG00000169181 | GSG1L | 16.75 | 75 | 0.038361062 | 0.000357992 | 0.04523757 |
| ENSG00000198075 | SULT1C4 | 12.5 | 50.75 | 1.193267385 | 0.000373053 | 0.04672937 |
| ENSG00000165186 | PTCHD1 | 116 | 301 | 0.59937543 | 0.000390971 | 0.04858816 |
| ENSG00000231811 | RPL36AP35 | 72 | 30.5 | -1.410768434 | 0.000397497 | 0.04901325 |
| ENSG00000129675 | ARHGEF6 | 300 | 288.5 | -0.523660143 | 0.000402461 | 0.04924062 |
| ENSG00000106565 | TMEM176B | 74.75 | 56.25 | -0.880674525 | 0.000424806 | 0.05157466 |
| ENSG00000128052 | KDR | 66 | 517.5 | 0.017549658 | 0.000432156 | 0.0520666 |
| ENSG00000125848 | FLRT3 | 82.75 | 223.5 | 0.634538969 | 0.000446319 | 0.05336556 |
| ENSG00000111859 | NEDD9 | 85 | 260.25 | 0.761798286 | 0.000458592 | 0.05442074 |
| ENSG00000148204 | CRB2 | 11.75 | 79.75 | 0.026431803 | 0.000465466 | 0.05482423 |
| ENSG00000280285 | AC108215.1 | 30.75 | 15.25 | -1.494701227 | 0.000480114 | 0.05613063 |
| ENSG00000184216 | IRAK1 | 65.75 | 54 | -0.731070399 | 0.000489958 | 0.05686031 |
| ENSG00000087245 | MMP2 | 38 | 174.25 | 0.043401601 | 0.000503278 | 0.05797982 |
| ENSG00000100815 | TRIP11 | 122.75 | 303.25 | 0.513601324 | 0.00054065 | 0.06113562 |
| ENSG00000146833 | TRIM4 | 5.5 | 0.25 | -3.927054485 | 0.000540401 | 0.06113562 |
| ENSG00000241644 | RPS23P6 | 7.75 | 39 | 1.301122224 | 0.000542291 | 0.06113562 |
| ENSG00000105290 | APLP1 | 53.75 | 186.5 | 0.993973783 | 0.000580278 | 0.06486219 |
| ENSG00000142089 | IFITM3 | 202.25 | 569.25 | 0.74077742 | 0.000587676 | 0.06486219 |
| ENSG00000143416 | SELENBP1 | 53.25 | 38.75 | -0.878599546 | 0.000587546 | 0.06486219 |
| ENSG00000165238 | WNK2 | 1143.25 | 1279.75 | -0.372300109 | 0.000598594 | 0.06560835 |
| ENSG00000198846 | TOX | 111.75 | 257.75 | 0.465106829 | 0.000670077 | 0.07282824 |
| ENSG00000286039 |  | 202.25 | 177.75 | -0.620889169 | 0.000673695 | 0.07282824 |
| ENSG00000287661 |  | 32 | 12.25 | -1.506911004 | 0.000687284 | 0.07379184 |
| ENSG00000113805 | CNTN3 | 3 | 27 | 0.019646465 | 0.000694462 | 0.07405877 |
| ENSG00000119900 | OGFRL1 | 52.75 | 168.25 | 0.834263969 | 0.000736648 | 0.07803032 |
| ENSG00000016082 | ISL1 | 56.75 | 156.75 | 0.678196945 | 0.00076597 | 0.08059531 |
| ENSG00000168824 | NSG1 | 12.75 | 72.75 | 0.026775494 | 0.000778814 | 0.08140416 |
| ENSG00000065320 | NTN1 | 46.5 | 32.25 | -0.909427523 | 0.000790509 | 0.08154647 |
| ENSG00000181072 | CHRM2 | 704 | 1503.75 | 0.410718536 | 0.000785416 | 0.08154647 |
| ENSG00000179862 | CITED4 | 62.75 | 50.25 | -0.742624485 | 0.000796283 | 0.08160869 |
| ENSG00000157827 | FMNL2 | 94.5 | 252 | 0.601179854 | 0.000817612 | 0.08325401 |
| ENSG00000170381 | SEMA3E | 4.5 | 33.5 | 0.020796257 | 0.000860267 | 0.08703584 |
| ENSG00000197747 | S100A10 | 41 | 301.5 | 0.019160701 | 0.000883571 | 0.08882419 |
| ENSG00000141756 | FKBP10 | 216.75 | 493.5 | 0.449848569 | 0.000921307 | 0.09203158 |
| ENSG00000107521 | HPS1 | 147.5 | 153.75 | -0.450522184 | 0.000930536 | 0.09236749 |
| ENSG00000155966 | AFF2 | 29.25 | 103.25 | 0.040805948 | 0.000936374 | 0.09236749 |
| ENSG00000054938 | CHRDL2 | 2.25 | 28.25 | 0.01447017 | 0.000974478 | 0.09536758 |
| ENSG00000156381 | ANKRD9 | 184.75 | 178 | -0.518224936 | 0.000978873 | 0.09536758 |
| ENSG00000240583 | AQP1 | 845.25 | 2169.5 | 0.548704979 | 0.000993507 | 0.09619956 |
| ENSG00000123689 | G0S2 | 140.75 | 378.5 | 0.696420022 | 0.001020407 | 0.09765018 |
| ENSG00000165300 | SLITRK5 | 7.75 | 36.75 | 1.225115597 | 0.001020863 | 0.09765018 |
| ENSG00000158258 | CLSTN2 | 564.25 | 1409.5 | 0.571027493 | 0.001036613 | 0.09797347 |
| ENSG00000172201 | ID4 | 22.5 | 75.25 | 0.049580782 | 0.001036658 | 0.09797347 |
| ENSG00000157600 | TMEM164 | 401 | 430.25 | -0.410838656 | 0.001045955 | 0.09826377 |
| ENSG00000066032 | CTNNA2 | 5 | 33 | 0.021327659 | 0.00106546 | 0.0984061 |
| ENSG00000157680 | DGKI | 502.75 | 1132 | 0.467273908 | 0.001066175 | 0.0984061 |
| ENSG00000173376 | NDNF | 26 | 14.25 | -1.282081645 | 0.00106447 | 0.0984061 |
| ENSG00000182718 | ANXA2 | 1541.75 | 3309.25 | 0.356936405 | 0.001089411 | 0.09996616 |

**Supplementary Table 4. *RRAGD*^WT/p.S76L^ hiPSC-CMs differentially expressed genes vs. DCM.** List of genes differentially expressed in our bulk RNA-seq data that overlapped with genes specific to DCM patients in Sweet *et al.* Log2FC: Log2 fold change, padj: adjusted p-value.

| Gene ID | hgnc_symbol | Mean count WT | Mean count S76L | Log2FC | p-value | padj |
| --- | --- | --- | --- | --- | --- | --- |
| ENSG00000136383 | ALPK3 | 2056.75 | 500 | -2.6435195 | 4.45E-97 | 7.02E-93 |
| ENSG00000145741 | BTF3 | 494 | 1435.75 | 0.91179882 | 1.44E-12 | 3.78E-09 |
| ENSG00000152583 | SPARCL1 | 9.25 | 132.25 | 3.21368122 | 4.61E-12 | 1.04E-08 |
| ENSG00000134716 | CYP2J2 | 756.75 | 590.75 | -0.9207944 | 1.23E-10 | 1.94E-07 |
| ENSG00000125730 | C3 | 3.25 | 152.5 | 0.02475256 | 2.54E-09 | 0.00000287 |
| ENSG00000197614 | MFAP5 | 8 | 113 | 0.04031051 | 5.85E-08 | 0.000045 |
| ENSG00000105894 | PTN | 706.75 | 1772.25 | 0.69459123 | 2.14E-07 | 0.0001251 |
| ENSG00000077009 | NMRK2 | 59.75 | 23.25 | -1.8596059 | 2.39E-07 | 0.0001259 |
| ENSG00000175206 | NPPA | 3996 | 3282.5 | -0.8292106 | 3.16E-07 | 0.00016114 |
| ENSG00000160808 | MYL3 | 9677.5 | 7296.25 | -0.9133959 | 4.73E-07 | 0.00023326 |
| ENSG00000100461 | RBM23 | 273.75 | 249.25 | -0.6603165 | 0.0000035 | 0.00145271 |
| ENSG00000197893 | NRAP | 514 | 186.75 | -2.0533279 | 0.00000448 | 0.00176707 |
| ENSG00000197442 | MAP3K5 | 129.5 | 118 | -0.6690651 | 0.0000254 | 0.00635819 |
| ENSG00000137936 | BCAR3 | 68 | 221.25 | 1.00976701 | 0.0000355 | 0.00861396 |
| ENSG00000169908 | TM4SF1 | 1.5 | 32 | 3.1841176 | 0.0000386 | 0.00909285 |
| ENSG00000125378 | BMP4 | 20.5 | 102 | 1.43652945 | 0.0000588 | 0.01271258 |
| ENSG00000109472 | CPE | 87 | 257.5 | 0.76355562 | 0.0000726 | 0.01468722 |
| ENSG00000176658 | MYO1D | 96.25 | 271.75 | 0.77744044 | 0.0000771 | 0.01514495 |
| ENSG00000171476 | HOPX | 329.5 | 262 | -0.8969448 | 0.00010862 | 0.01970495 |
| ENSG00000162630 | B3GALT2 | 290 | 692.75 | 0.59723795 | 0.00015015 | 0.02443134 |
| ENSG00000088992 | TESC | 103.75 | 283.75 | 0.73006375 | 0.0001753 | 0.02695263 |
| ENSG00000154330 | PGM5 | 727.5 | 830.75 | -0.3454609 | 0.0002774 | 0.0392433 |
| ENSG00000068976 | PYGM | 451.75 | 406.5 | -0.5920419 | 0.00028417 | 0.03934234 |
| ENSG00000143324 | XPR1 | 1325.5 | 2871.5 | 0.42070442 | 0.00034581 | 0.04502721 |
| ENSG00000182197 | EXT1 | 537.25 | 1268 | 0.50694426 | 0.00034281 | 0.04502721 |
| ENSG00000106565 | TMEM176B | 74.75 | 56.25 | -0.8806745 | 0.00042481 | 0.05157466 |
| ENSG00000128052 | KDR | 66 | 517.5 | 0.01754966 | 0.00043216 | 0.0520666 |
| ENSG00000111859 | NEDD9 | 85 | 260.25 | 0.76179829 | 0.00045859 | 0.05442074 |
| ENSG00000184216 | IRAK1 | 65.75 | 54 | -0.7310704 | 0.00048996 | 0.05686031 |
| ENSG00000087245 | MMP2 | 38 | 174.25 | 0.0434016 | 0.00050328 | 0.05797982 |
| ENSG00000105290 | APLP1 | 53.75 | 186.5 | 0.99397378 | 0.00058028 | 0.06486219 |
| ENSG00000142089 | IFITM3 | 202.25 | 569.25 | 0.74077742 | 0.00058768 | 0.06486219 |
| ENSG00000143416 | SELENBP1 | 53.25 | 38.75 | -0.8785995 | 0.00058755 | 0.06486219 |
| ENSG00000165238 | WNK2 | 1143.25 | 1279.75 | -0.3723001 | 0.00059859 | 0.06560835 |
| ENSG00000179862 | CITED4 | 62.75 | 50.25 | -0.7426245 | 0.00079628 | 0.08160869 |
| ENSG00000197747 | S100A10 | 41 | 301.5 | 0.0191607 | 0.00088357 | 0.08882419 |
| ENSG00000141756 | FKBP10 | 216.75 | 493.5 | 0.44984857 | 0.00092131 | 0.09203158 |
| ENSG00000123689 | G0S2 | 140.75 | 378.5 | 0.69642002 | 0.00102041 | 0.09765018 |
| ENSG00000182718 | ANXA2 | 1541.75 | 3309.25 | 0.35693641 | 0.00108941 | 0.09996616 |
