# Supplementary figures and images for: RRAGD p.(Ser76Leu) Variant Causes Dysregulated Expression of Muscle Development and Cytoskeleton Genes in Cardiomyocytes
Source: FASEB J. 2026 Jun 22;40(12):e72070. doi: 10.1096/fj.202501099RR (PMC13285898; doi:10.1096/fj.202501099RR)

Suppl. Fig. 1: Effects of mTOR inhibition by rapamycin in HeLa RagD-S76L cells

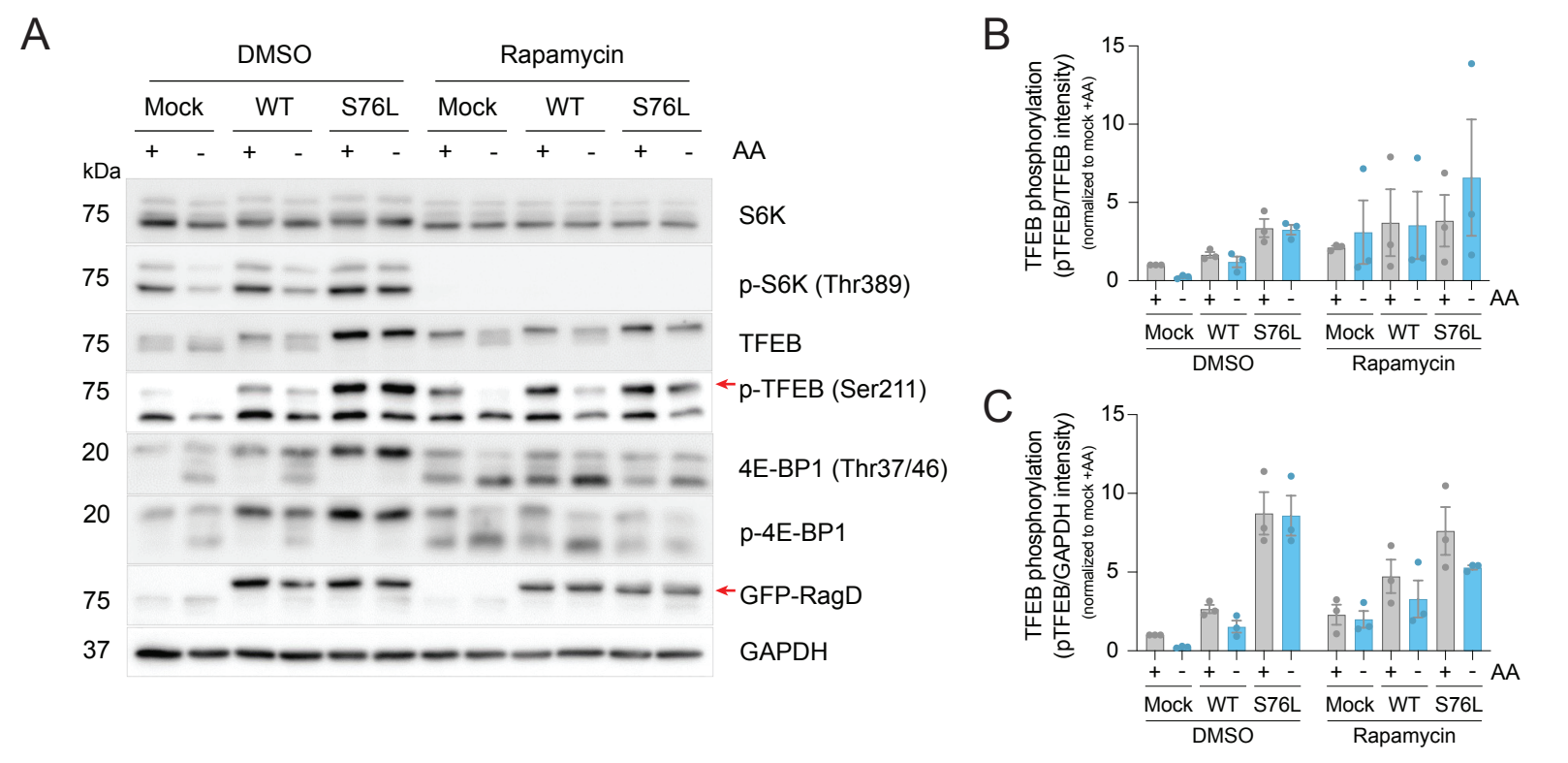

Supplement: Supplementary file 2 — Figure S1: mTOR inhibition by rapamycin suppressed S6K and 4E‐BP1 phosphorylation but not TFEB in RRAGD p.(Ser76Leu) T‐REx HeLa cells. (A‐C) T‐REx HeLa cells stably overexpressing GFP (mock), GFP‐RRAGD WT (WT), and GFP‐RRAGD p.(Ser76Leu) (S76L) were exposed to amino acids stimulation using amino acids‐containing medium (+AA, gray bars) or amino acids‐deprived medium (−AA, blue bars) and rapamycin or DMSO. (A) Representative immunoblots of S6K, p‐S6K, TFEB, p‐TFEB, 4E‐BP1, p‐4E‐BP1, GFP, and GAPDH following treatment. (B, C) Quantification of (B) phosphorylated TFEB/total TFEB ratio and (C) phosphorylated TFEB/GAPDH ratio (mean ± SEM from three independent experiments, normalized to the DMSO mock +AA condition). Two‐way ANOVA followed by Šídák's multiple comparison test where the effects of DMSO and rapamycin treatment were compared within each genotype, and each amino acids treatment group. The normality of the distribution was tested using a Q‐Q plot. [file FSB2-40-e72070-s004.pdf]

Suppl. Fig. 2: CRISPR-Cas9-mediated generation of hiPSCs

A

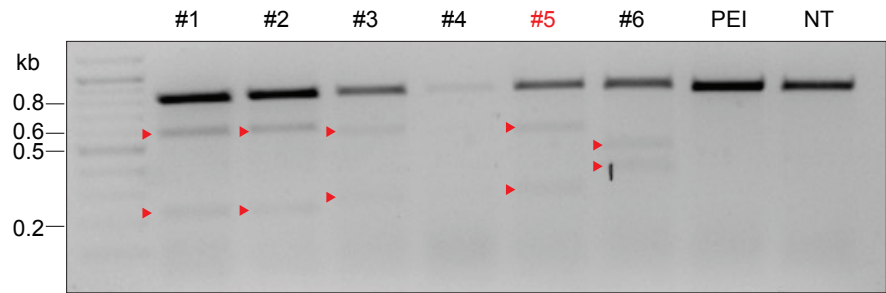

B

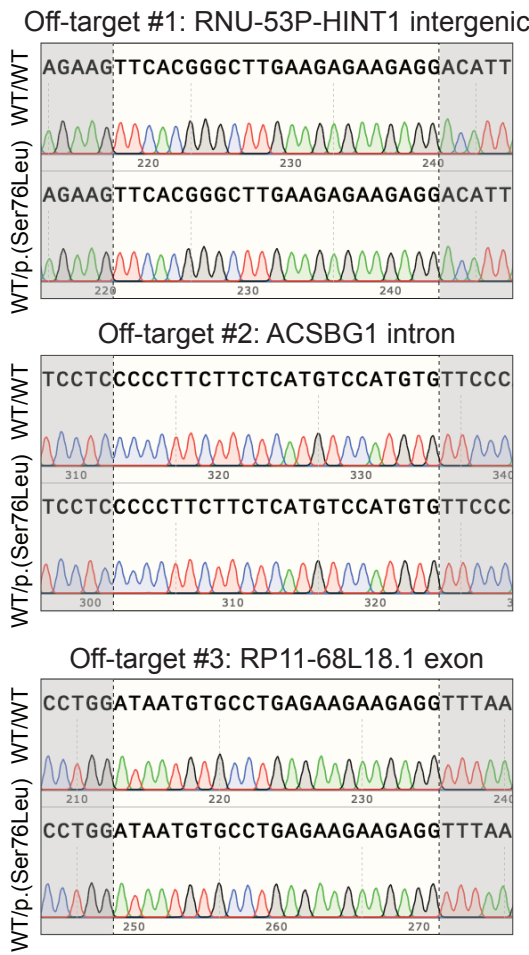

C

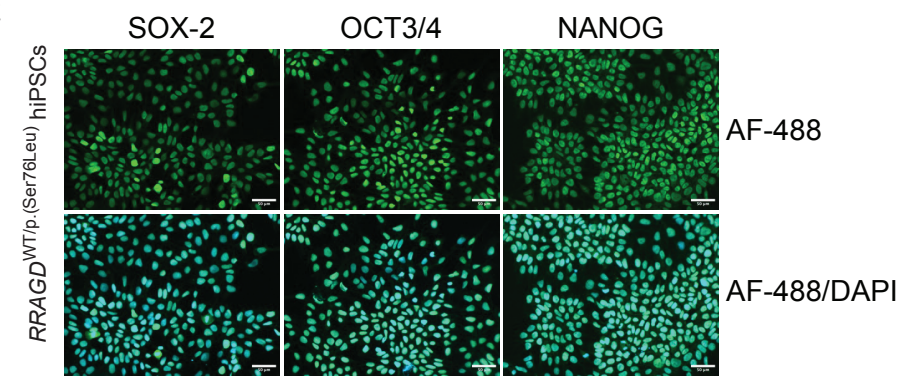

D

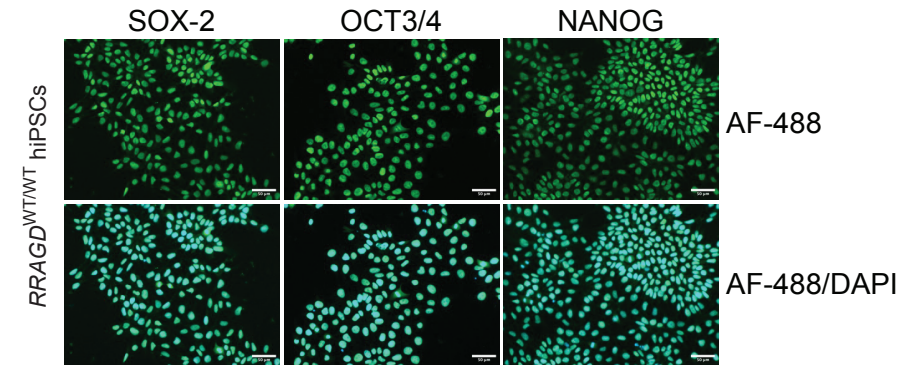

E

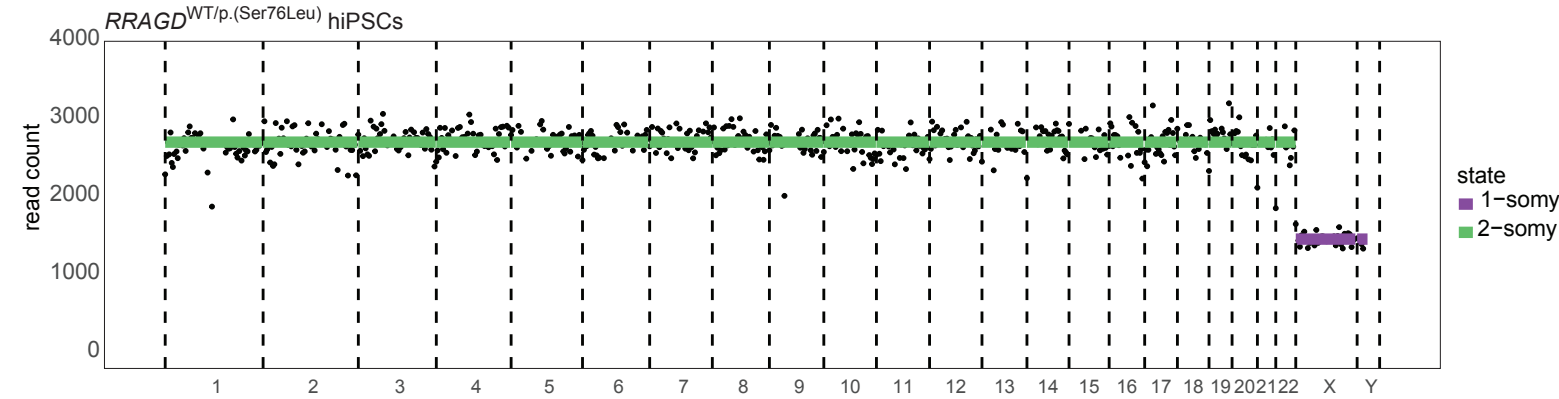

F

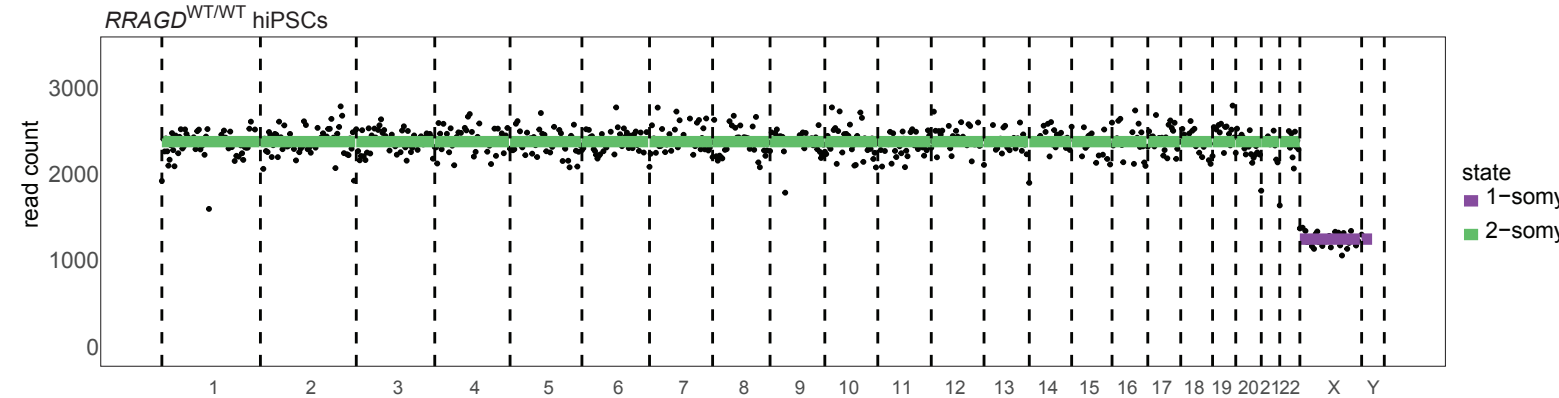

Supplement: Supplementary file 3 — Figure S2: CRISPR‐Cas9‐mediated generation of RRAGD WT/WT and RRAGD WT/p.(Ser76Leu) hiPSCs. (A) T7 endonuclease assay in HEK293 cells gDNA transiently transfected with either one of the six gRNAs, only transfection agent (i.e., PEI), or cells without any treatment (non‐treated or NT). gRNA #5 was picked for subsequent steps. Red arrows indicate DNA fragments cut by T7 endonuclease. (B) Sanger sequencing results of the top three predicted off‐target sites on gRNA #5. (C, D) Representative immunofluorescence images of pluripotency markers SOX‐2, OCT3/4, and NANOG (green), and DAPI counterstain (blue) in (C) RRAGD WT/p.(Ser76Leu) and (D) RRAGD WT/WT hiPSCs clones. Scale bars indicate 20 μm. (E, F) Karyo‐sequencing profiles of (E) RRAGD WT/p.(Ser76Leu) and (F) RRAGD WT/WT hiPSCs clones. [file FSB2-40-e72070-s001.pdf]

Suppl. Fig. 3: bulk RNA sequencing data

A

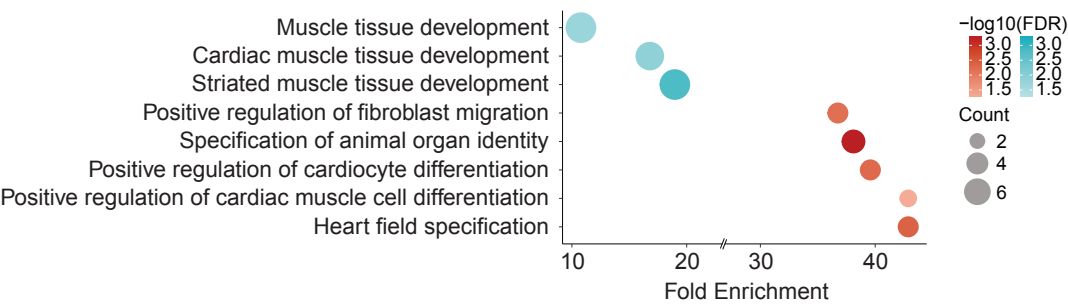

B

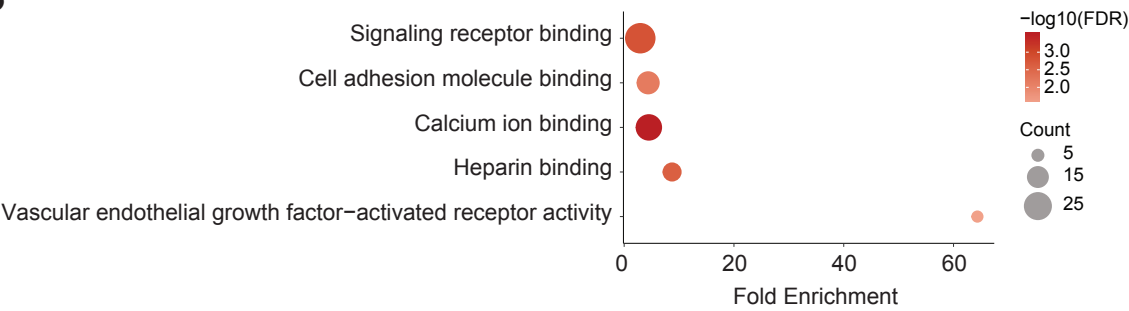

C

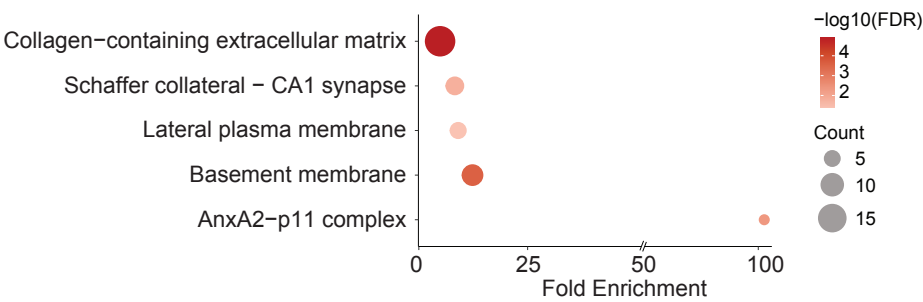

Supplement: Supplementary file 4 — Figure S3: Enriched gene ontology (GO) terms within the bulk RNA‐seq differentially expressed gene list. (A–C) Bubble plots of top enriched (A) biological process, (B) molecular function, and (C) cellular component GO terms in RRAGD WT/p.(Ser76Leu) hiPSC‐CMs. The terms have been filtered for those relevant in cardiomyocytes. The ranking of the terms was based on fold enrichment. The intensity of the bubble color is based on −log10 false discovery rate (FDR); red shows upregulated pathways, and blue shows downregulated pathways. The bubble size indicates the number of genes in each pathway (count). [file FSB2-40-e72070-s003.pdf]

Suppl. Fig. 4: RNA-seq validation

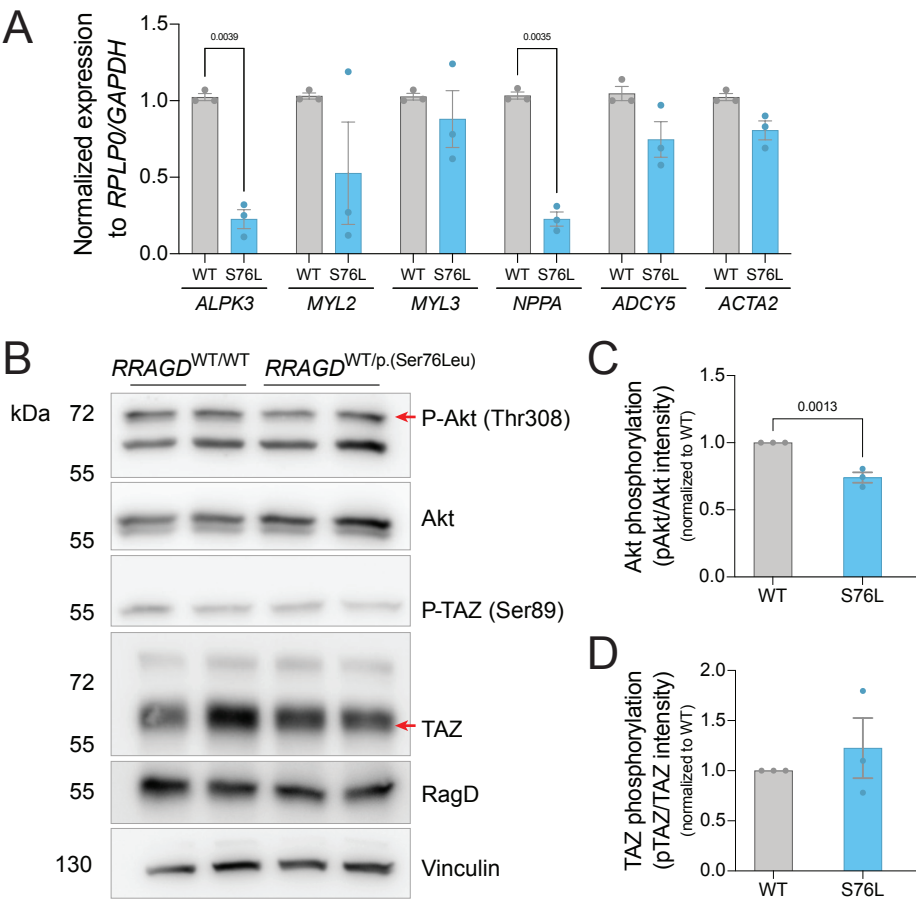

Supplement: Supplementary file 5 — Figure S4: Validation of bulk RNA‐seq. (A) mRNA expression of differentially expressed genes associated with cardiomyopathies in RRAGD WT/WT (WT; gray bars) and RRAGD WT/p.(Ser76Leu) (S76L; blue bars) hiPSC‐CMs. Mean ± SEM from three independent differentiations. Two‐way ANOVA followed by Šídák's multiple comparison test was performed. The normality of the distribution was tested using a Q‐Q plot. (B) Representative immunoblots of p‐Akt, Akt, p‐TAZ, TAZ, RagD, and vinculin in RRAGD WT/WT and RRAGD WT/p.(Ser76Leu) hiPSC‐CMs. (C‐D) Quantification of (C) Akt phosphorylation and (D) TAZ phosphorylation immunoblots in RRAGD WT/WT (WT; gray bars) and RRAGD WT/p.(Ser76Leu) (S76L; blue bars) hiPSC‐CMs. Mean ± SEM from three independent differentiations, normalized to the WT. One‐tailed unpaired t‐test was performed. [file FSB2-40-e72070-s005.pdf]
